# Partial loss of Sorting Nexin 27 resembles age- and Down syndrome-associated T cell dysfunctions

**DOI:** 10.1186/s12979-023-00402-3

**Published:** 2024-01-02

**Authors:** Cristina Rodriguez-Rodriguez, Natalia González-Mancha, Ane Ochoa-Echeverría, Rosa Liébana, Isabel Merida

**Affiliations:** Department of Immunology and Oncology, Spanish National Centre for Biotechnology (CNB-CSIC), UAM Campus de Cantoblanco, Darwin 3, 28049 Madrid, Spain

**Keywords:** Aging, Down syndrome, Cytokines, Lymphocyte, Inflammation, SNX27

## Abstract

**Background:**

Sorting Nexin 27 (SNX27)-retromer complex facilitates cargo recycling from endosomes to the plasma membrane. SNX27 downregulation in neurons, as the result of Trisomy 21 (T21), has been linked with cognitive deficits due to impairment of AMPA and NMDA receptor recycling. Studies in human T cell lines likewise demonstrated that SNX27 regulates the correct delivery of cargoes to the immune synapse limiting the activation of pro-inflammatory pathways. Nevertheless, the physiological consequences of partial SNX27 loss in T cell homeostasis are still unclear.

**Results:**

In this study, we have explored the consequences of T cell specific partial SNX27 downregulation in mice. T cells with partial SNX27 deficiency show a marked deficit in the CD4^+^ T cell pool, a hallmark of aging in mice and humans, and a well-characterized comorbidity of individuals with Down syndrome (DS). When analyzed ex vivo, CD4^+^ T cells with partial SNX27 deletion demonstrate enhanced proliferation but diminished IL-2 production. In contrast, the CD8^+^ population show enhanced expression of pro-inflammatory cytokines and lytic enzymes.

**Conclusions:**

This mouse model supports the relevance of SNX27 in the organization of the immune synapse, previously described in cell lines, as well as in the control of T cell homeostasis. Individuals with DS experiment an acceleration of the aging process, which particularly affects the immune and central nervous systems. Thus, we hypothesize that reduced SNX27 expression in DS could contribute to the dysregulation of these systems and further research in SNX27 will shed light on the molecular factors underlying the phenotypes observed in people with DS and its contribution to aging.

**Supplementary Information:**

The online version contains supplementary material available at 10.1186/s12979-023-00402-3.

## Background

SNX27 is an endosomal, sorting protein that participates in the PDZ (Postsynaptic density 95/disc large/zonula occludens 1)-dependent trafficking of transmembrane proteins including receptors, channels, and amino acid transporters [1–5]. In association with the retromer and the WASH complex, SNX27 maintains the correct expression at the cell surface of its cargoes. SNX27 null mice are small and die early after birth [4], whereas heterozygous mice show Down Syndrome (DS)-like cognitive impairment [6], which was recovered after SNX27 reconstitution in these mice, suggesting that one single gene can restore synaptic transmission and cognitive functions [6, 7]. In addition, SNX27 partial loss enhances amyloid precursor protein processing [8], linking defects in SNX27 expression to Alzheimer´s disease.

Aging is characterized by a progressive decline in physiological functions including the immune response and cognitive abilities [9]. The cognitive deterioration and early onset of Alzheimer´s disease associated with Trisomy 21 (T21) suggest accelerated aging [10]. Premature aging of the immune system in individuals with DS corresponds with their higher susceptibility to bacterial infections, hematological malignancies, and autoimmune disorders [11, 12]. Key features of the aging adaptive immune response include reduced capacity to respond to antigens and loss of naïve T cell populations [13, 14]. These defects are commonly associated with a low grade of chronic inflammation (inflammaging) that is an important driver of aging-related diseases [15].

In T lymphocytes, SNX27 participates in the control of cargo delivery to the immune synapse (IS). The main PDZ binding cargoes of SNX27 in T cells are cytosolic signaling proteins that regulate lipid composition and cytoskeletal remodeling [16], in contrast to the transmembrane receptors that are the main SNX27 cargoes in the brain [4, 5]. SNX27 null mice and SNX27-silenced human T cell lines show enhanced activation of nuclear factor κB (NFκB)-dependent transcription with concomitant downregulation of mammalian target of rapamycin (mTOR) activation [17]. This phenotype partially resembled that described in the immune system of people with DS [18–20], suggesting that a deficit in SNX27 could result in the alteration of their immune response. More recent studies demonstrated that partial SNX27 silencing in human T cell lines results in defective polarization of the microtubule organizing center (MTOC) [21]. These results provide a direct correlation between SNX27-dependent control of protein traffic and the correct organization of the immune synapse.

In this study, we generated mice heterozygous for SNX27 in the T cell compartment and characterized primary T cell development and function. We present evidence that the immunological phenotype of SNX27 mice resembles in many ways age-associated changes of the adaptive immune system, both under steady state conditions as well as after in vitro stimulation. More precisely, these mice show reduced percentage of CD4^+^ T cells under steady-state conditions, as well as enhanced relative numbers of CD8^+^ T cells. Upon activation, CD4^+^ T cells displayed diminished IL-2 production that contrasted with their enhanced proliferation. As previously observed in human T cell lines, partial SNX27 deficiency in CD4 T^+^ cells impaired MTOC synaptic recruitment upon antigenic triggering, suggesting substantial defects at the early stages of activation. In contrast, ex vivo differentiated cytotoxic T lymphocytes (CTL) showed enhanced production of inflammatory cytokines and lytic enzymes without defects in their killing ability. Overall, our studies demonstrate that partial SNX27 loss has a profound effect on T cell functions and suggest that its downregulation could contribute to the immune alterations associated with DS and resembles premature aging.

## Results

### Generation of mice with partial SNX27 deficiency in the T cell compartment

**Fig. 1:**
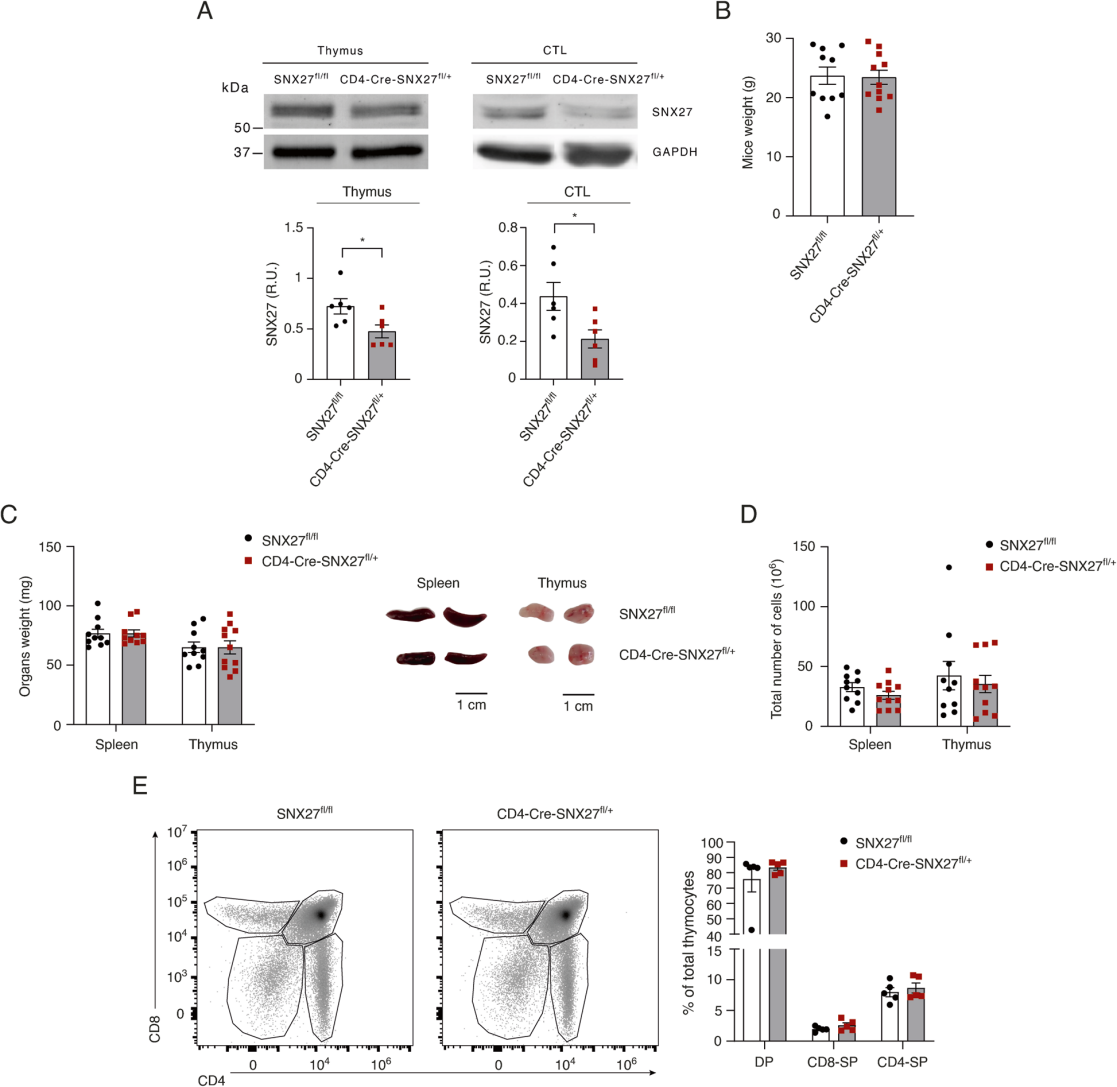
Partial deletion of SNX27 in CD4-Cre-SNX27^fl/+^ mice does not alter lymphoid organ size and cellularity. **A-E** SNX27^fl/fl^ or CD4-Cre-SNX27^fl/+^ mice were sacrificed. **A** Expression of SNX27 in thymus and ex vivo-differentiated CTL was examined by Western Blot, confirming partial depletion of SNX27. GAPDH was used as a loading control. A representative blot and the quantification of SNX27 levels in thymus and CTL are shown (*n* = 6 mice). Values were normalized against GAPDH. Data are shown as mean ± SEM; *p < 0.05; unpaired *t*-test. **B** Body weight comparison among age-matched pairs is shown. **C** Spleens and thymus were extracted and weighted. Scale bar = 1 cm. **D** Cells from these organs were extracted and total cellularity was analyzed using a cell counter. **E** Thymocytes were stained for the indicated surface markers and analyzed by flow cytometry (gating strategy shown in Suppl Fig. 1). The percentage of total double positive (DP; CD8^+^CD4^+^) and single positive (SP; CD8^+^CD4^−^ or CD8^−^CD4^+^) thymocytes was calculated (right graph). Representative flow cytometry plots are shown (left panels). Data shown as mean ± SEM of 10–11 mice in (**B-D**) and 5 mice in (**E**). Significance in (**B**) was determined by unpaired *t*-test, and two-way ANOVA/Bonferroni post-test was used for multiple comparisons in (**C-E**); ns *p* > 0.05. *CTL* Cytotoxic T lymphocyte, *SNX* Sorting nexin, *DP* Double positive, *SP* Single positive, *SEM* Standard error of the mean

SNX27 is ubiquitously expressed, complicating the analysis of its role on T cell functions even in heterozygous mice. Therefore, we generated a new transgenic mouse line bearing T cell-specific SNX27 silencing. SNX27 protein expression was depleted from the thymic double positive (DP) stage onwards by crossing mice displaying SNX27 floxed alleles (SNX27^flox/flox^) with CD4^+^-Cre transgenic mice. The generated SNX27^+/-^ heterozygote mice (from now on CD4-Cre-SNX27^fl/+^) were kept in a C57BL/6 genetic background. SNX27^flox/flox^ mice were used as controls (named SNX27^fl/fl^). To examine that the inserted SNX27 floxed alleles were susceptible to CD4-Cre cleavage in vivo, we compared SNX27 expression in thymus and ex vivo-differentiated CTL between SNX27^fl/fl^ and CD4-Cre-SNX27^fl/+^ mice. As expected, SNX27 was reduced in cell lysates from thymus and CTL in CD4-Cre-SNX27^fl/+^ mice compared to SNX27^fl/fl^ ones (Fig. 1A). This confirmed the specific depletion of SNX27 in our mouse model. Consistent with reported data from heterozygous SNX27^+/-^ mice [4], SNX27^fl/fl^ and CD4-Cre-SNX27^fl/+^ age-paired mice displayed similar body weight (Fig. 1B). No differences were found either when analyzing spleen and thymus weights and sizes (Fig. 1C), which correlated with comparable total cellularity in these organs (Fig. 1D). The analysis of thymic populations revealed no major differences in the differentiation of CD4^+^ and CD8^+^ pools (Fig. 1E) (gating strategy shown in Suppl Fig. 1). These results indicate that SNX27-specific deficiency in CD4-Cre-SNX27^fl/+^ mice does not affect lymphoid organs development.

### Partial SNX27 deficiency in T cells leads to reduced relative numbers of CD4^+^ T lymphocytes in circulating blood and in secondary lymphoid organs

**Fig. 2:**
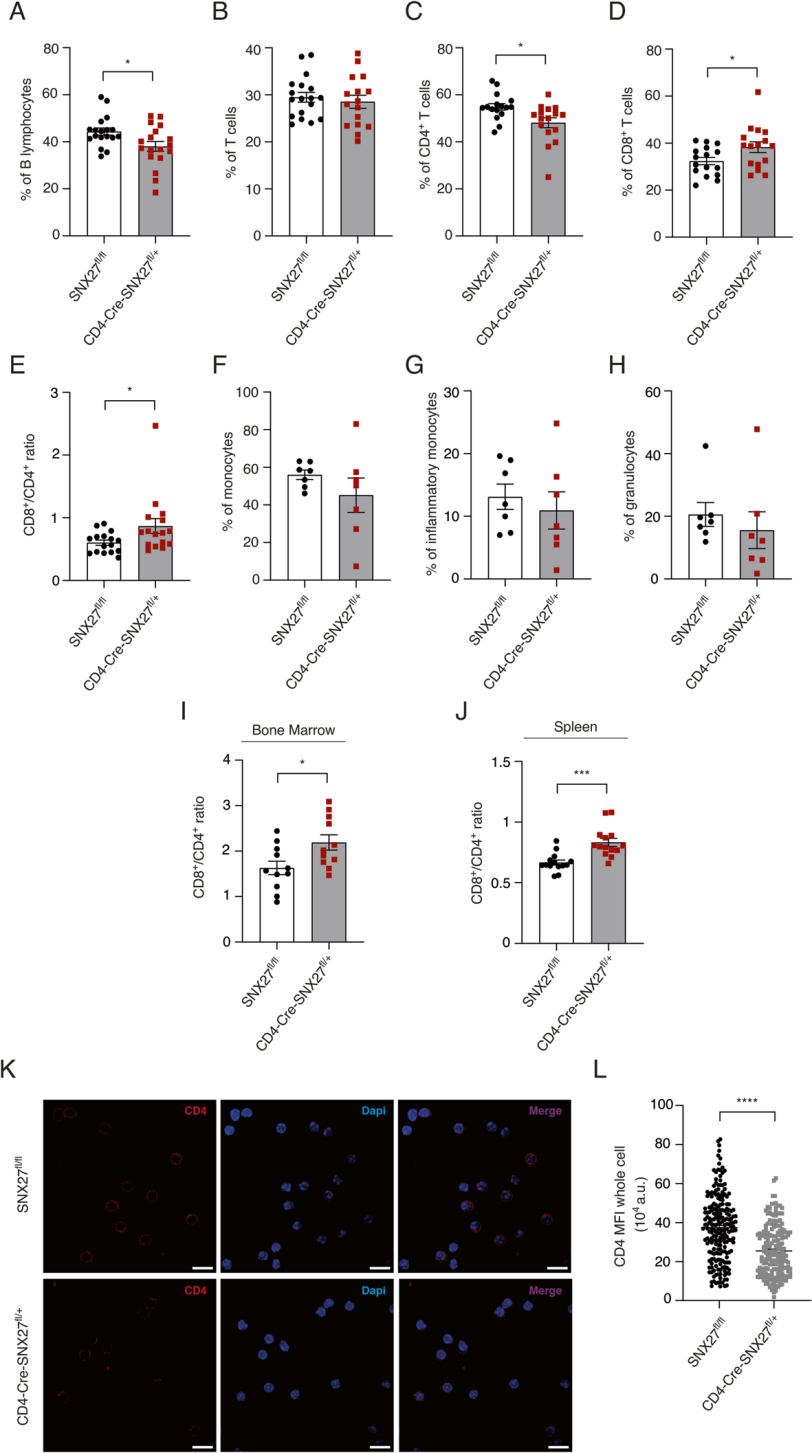
Partial SNX27 deficiency in CD4-Cre-SNX27^fl/+^ mice dysregulates circulating lymphocyte populations. **A**-**H** Aliquots of whole blood from SNX27^fl/fl^ and CD4-Cre-SNX27^fl/+^ were incubated with a mix of LIVE/DEAD violet fixable cell stain and the following surface markers: CD45, CD3, B220, CD8, CD4, Ly6C, and Ly6G (20 min, RT). Versalyte solution was then added to lyse red cells (10 min, RT, darkness). Afterwards, flow cytometry analysis of the distinct populations was performed. Histograms depicting percentage of (**A**) B cells (CD45^+^ CD3^−^ B220^+^), (**B**) T cells (CD45^+^ CD3^+^ B220^−^), (**C**) CD4^+^ and (**D**) CD8^+^ (CD45^+^ B220^−^ CD3^+^), (**E**) the ratio between CD8^+^ and CD4^+^ cells, (**F**) monocytes (CD45^+^ CD3^−^ B220^−^ Ly6C^+^), (**G**) inflammatory monocytes (CD45^+^ CD3^−^ B220^−^ Ly6C^+^ high), and (**H**) granulocytes (CD45^+^ CD3^−^ B220^−^ Ly6G^+^) are shown. Gating strategy is shown in Suppl Fig. 3. Data shown as mean ± SEM; *p < 0.05; unpaired *t*-test; *n* = 12 mice in (**A**-**E**), and *n* = 7 mice in (**F**–**H**). **I, J** CD8^+^/CD4^+^ ratio in bone marrow (**I**) and spleen (**J**) is shown. Gating strategies used are depicted in Suppl Fig. 4. Data shown as mean ± SEM; *p < 0.05; ***p < 0.001 unpaired *t*-test; *n* = 11 mice in (**I**) and *n *= 14 mice in (**J**). **K** Representative maximum intensity projections from confocal planes (z) of SNX27^fl/fl^ and CD4-Cre-SNX27^fl/+^ mice lymph nodes stained for CD4 in basal conditions. Nuclei were stained with DAPI (blue). Scale bar = 10 μm. **L** Quantification of CD4 expression measured as the signal intensity of the whole cell. Data are shown as ± SEM of an experiment (SNX27^fl/fl^ = 211 cells; CD4-Cre-SNX27^fl/+^ = 179 cells); unpaired *t-*test; ****p < 0.0001. *SNX* Sorting nexin, *RT* Room temperature, *SEM* Standard error of the mean

Blood analysis revealed normal hematological parameters (Suppl Fig. 2). Nonetheless, in-depth analysis using flow cytometry demonstrated slight but significant differences in the percentage of B cells in CD4-Cre-SNX27^fl/+^ mice compared to SNX27^fl/fl^ (Fig. 2A). Albeit the percentage of total T cells did not change in CD4-Cre-SNX27^fl/+^ mice compared to SNX27^fl/fl^ mice (Fig. 2B), a more detailed analysis revealed diminished percentage of CD4^+^ T cells (Fig. 2C) with a concomitant increase of CD8^+^ T cells (Fig. 2D), resulting in higher CD8^+^/CD4^+^ ratio (Fig. 2E). The rest of the analysis confirmed a similar percentage of classical and inflammatory monocytes as well as granulocytes in both mice genotypes (Fig. 2F-H). Analysis of bone marrow (Fig. 2I) and spleen (Fig. 2J) also showed increased CD8^+^/CD4^+^ ratios in CD4-Cre-SNX27^fl/+^ mice compared to those observed in control mice. This finding was confirmed by confocal microscopy after staining and quantification of CD4^+^ cells in lymph nodes from SNX27^fl/fl^ and CD4-Cre-SNX27^fl/+^ mice (Fig. 2K, L). These data resemble published data from individuals with DS and correspond with characteristic features in aging.

### CD4-Cre-SNX27^fl/+^ mice do not present major alterations relative to the memory CD4^+^ T cell pool under steady-state conditions

**Fig. 3:**
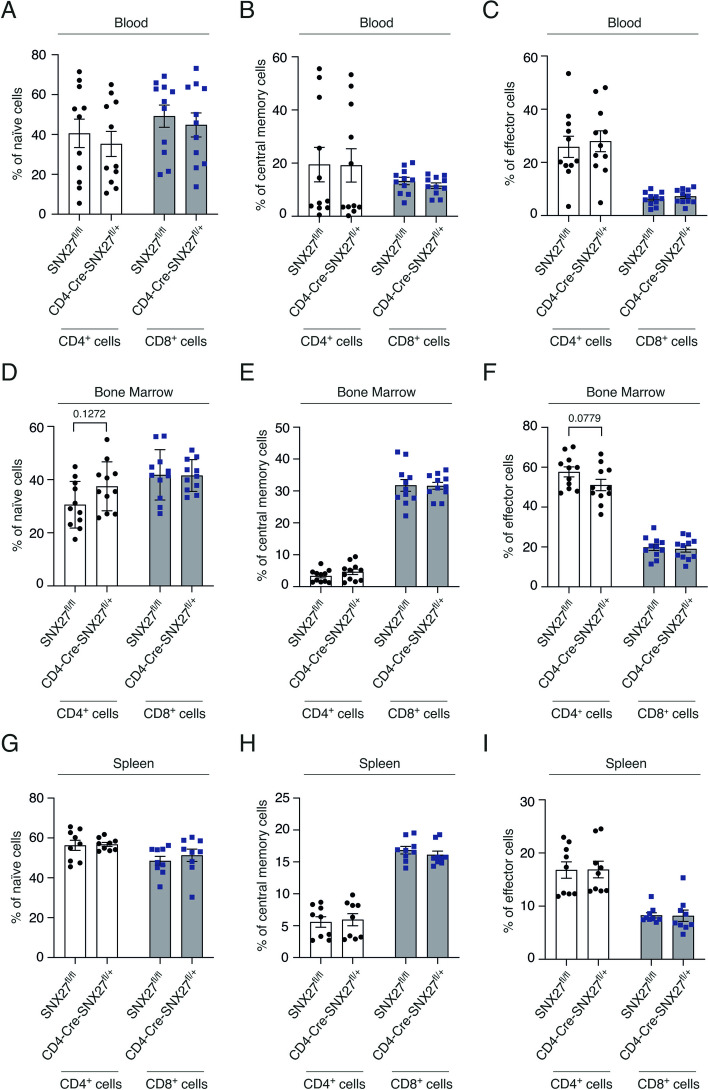
SNX27 partial depletion does not alter naïve, memory and effector T cell subsets. **A-I** Cells from SNX27^fl/fl^ and CD4-Cre-SNX27^fl/+^ mice were extracted from blood, bone marrow, and spleen. Graphs show the percentage of naïve (CD44^−^ CD62L^+^) (**A**, **D**, **G**), central memory (CD44^+^ CD62L^+^) (**B**, **E**, **H**), and effector (CD44^+^ CD62^−^) (**C**, **F**, **I**) cells among CD4^+^ and CD8^+^ T cell populations in (**A-C**) blood samples, (**D-F**) bone marrow cells, and (**G-I**) splenocytes. Gating strategies are shown in Suppl Fig. 5. Data shown as mean ± SEM; two-way ANOVA with Bonferroni post-test was used for multiple comparisons; ns *p* > 0.05; *n* = 11 mice in (**A-F**) and *n* = 9 mice in (**G-I**). *SNX* Sorting nexin, *SEM* Standard error of the mean

A deficit of CD4^+^ T cells could be the result of alterations in the naïve or memory pools. The relative numbers of naïve, central memory (Tcm) and effector/effector memory T cells were determined by the expression of the activation marker CD44 and the adhesion molecule CD62L by flow cytometry analysis [22]. Analysis of these populations in the different organs did not reveal significant differences in circulating blood (Fig. 3 A-C), bone marrow (Fig. 3D-F) or spleen (Fig. 3 G-I), which suggest that the reduction in CD4^+^ T cells expression in CD4-Cre-SNX27^fl/+^ mice is not due to an alteration in the distribution of naïve or memory cells populations.

### SNX27 partial deficiency in T cells enhances cell proliferation upon activation

**Fig. 4:**
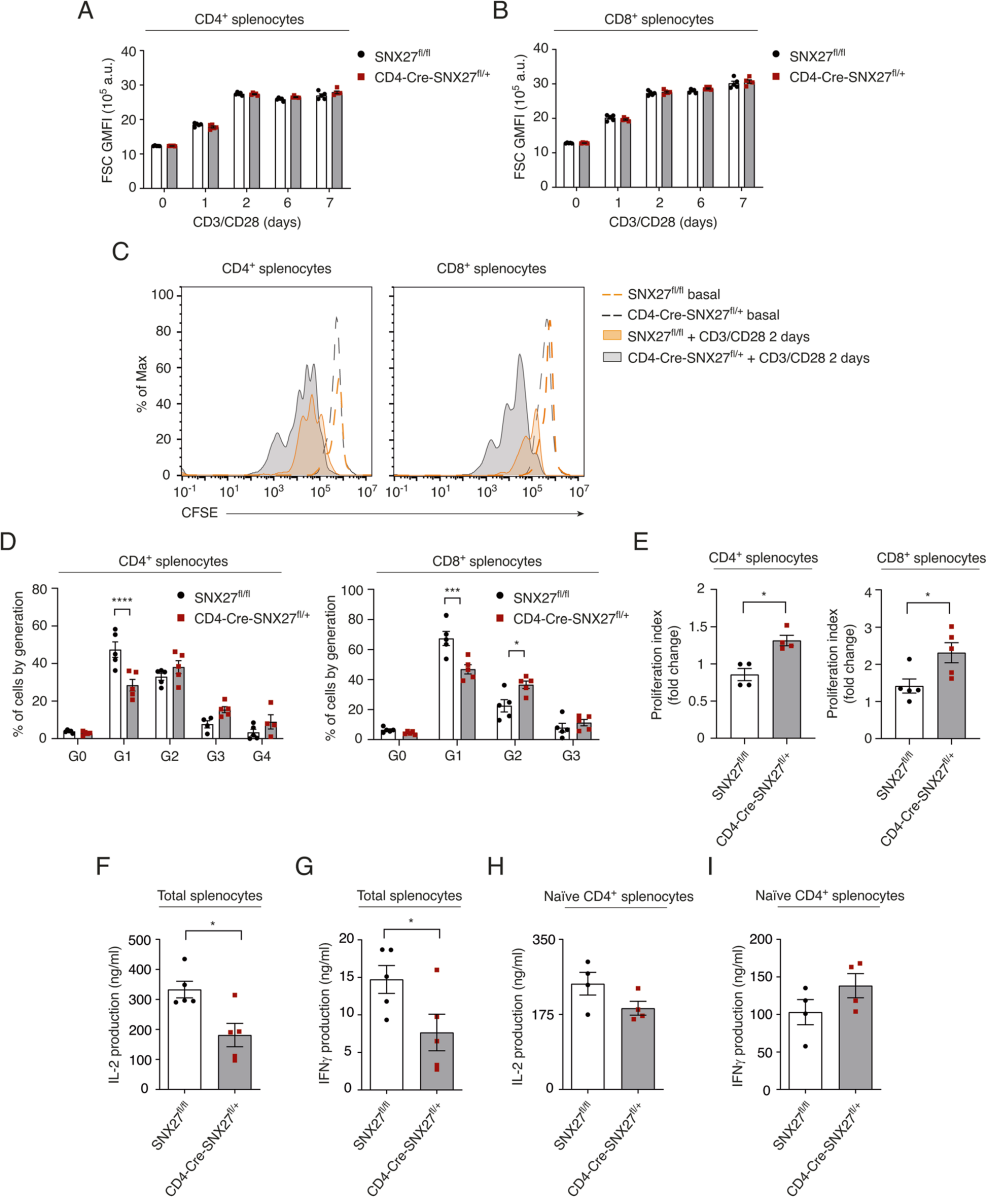
CD4-Cre-SNX27^fl/+^ splenocytes show normal growth and enhanced proliferation upon stimulation. **A, B** Splenocytes from SNX27^fl/fl^ or CD4-Cre-SNX27^fl/+^ mice were stimulated with plate-bound anti-CD3 (2.5 µg/ml) and soluble anti-CD28 (1.25 µg/ml) for 1, 2, 6 or 7 days. CD19^−^ CD3^+^ CD4^+^ and CD19^−^ CD3^+^ CD8^+^ T cells were gated by flow cytometry (gating strategy shown in Suppl Fig. 4). GMFI of the forward scatter, indicative of cell size, was calculated on these populations. Data are shown as mean ± SEM; two-way ANOVA with Bonferroni post-test was used for multiple comparisons; ns *p* > 0.05; *n* = 5 mice. **C-E** Splenocytes from SNX27^fl/fl^ or CD4-Cre-SNX27^fl/+^ mice were stained with CFSE. Labeled splenocytes were stimulated with plate-bound anti-CD3 (2.5 μg /mL) and soluble anti-CD28 (1.25 μg /mL) for 2 days. Cells were then stained with LIVE/DEAD violet fixable dye, and for CD19, CD3, CD4, and CD8 surface markers. CD19^−^ CD3^+^ CD4^+^ or CD19^−^ CD3^+^ CD8^+^ T cells were gated (gating strategy shown in Suppl Fig. 6). **C** CFSE dilution profiles of the gated populations are depicted. Representative flow cytometry plots are shown. **D, E** The percentage of cells by generation (**D**) and the proliferation index (**E**) are shown. Data shown as ± SEM; *p < 0.05; ***p < 0.001; ****p < 0.0001; two-way ANOVA with the Bonferroni post-test was used for multiple comparisons in (**D**) and unpaired *t*-test in (**E**); *n* = 5 mice in (**C-E**). **F, G** Total splenocytes or (**H, I**) isolated naïve CD4^+^ T cells from SNX27^fl/fl^ or CD4-Cre-SNX27^fl/+^ mice were stimulated 2 days with plate-bound anti-CD3 (2.5 µg/ml) and soluble anti-CD28 (1.25 µg/ml). Afterwards, the concentration of the depicted cytokines was assessed by ELISA. Data are shown as mean ± SEM of a representative experiment run in triplicate (*n* = 4–5 mice); *p < 0.05; unpaired *t-*test*. SNX* Sorting nexin, *GMFI* Geometric mean fluorescence intensity, *SEM* Standard error of the mean

Antigen recognition by naïve T cells elicits metabolic reprogramming to support a rapid phase of cell growth and subsequent proliferation. Previous studies demonstrate that T lymphocytes from SNX27^−/−^ mice display impaired growth upon TCR triggering [17], consistent with the observation that full *Snx27* deletion in mice resulted in smaller animals with reduced organ size [4, 6]. The size of splenocytes from SNX27^fl/fl^ or CD4-Cre-SNX27^fl/+^ mice stimulated ex vivo with anti-CD3/CD28 antibodies for 1, 2, 6, or 7 days was monitored by flow cytometry using the forward scatter (FSC) parameter. Cells reached their maximum size after 2 days of stimulation with no gross differences between SNX27^fl/fl^ and CD4-Cre-SNX27^fl/+^ T cells observed at any time point (Fig. 4A, B). This observation concurs with the normal size described in heterozygous SNX27^+/-^ mice [4] and suggests that defects in cell growth are associated to total SNX27 loss.

In contrast with defects in antigen-dependent growth, T cells from SNX27^−/−^ mice showed enhanced proliferation upon stimulation [17]. To determine if partial SNX27 loss affected proliferation, splenocytes from SNX27^fl/fl^ or CD4-Cre-SNX27^fl/+^ mice were stained with CFSE, and proliferating cells were identified by flow cytometry analysis of dye dilution upon stimulation. After 2 days of CD3/CD28 stimulation, we detected a higher proliferation rate in CD4^+^ CD4-Cre-SNX27^fl/+^ cells compared to SNX27^fl/fl^ cells that was even greater for the CD8^+^ T cell pool (Fig. 4C). Detailed quantification confirmed a lower percentage of CD4^+^ and CD8^+^ T cells at the initial stages of division, indicative of faster proliferation (Fig. 4D). A significative increase in the proliferation index was observed for CD4^+^ and CD8^+^T lymphocytes (Fig. 4E). These data indicate that partial SNX27 deficiency in T lymphocytes does not impair initial T cell growth but enhances proliferation.

Accelerated proliferation of CD4-Cre-SNX27^fl/+^ T cells could result from enhanced IL-2 production. Notwithstanding, the amount of IL-2 determined in the supernatant of CD4-Cre-SNX27^fl/+^ splenocytes 48 h after activation was significantly reduced compared to that determined in SNX27^fl/fl^ ones (Fig. 4F). Analysis of IFN γ also revealed a significant decrease, although the variability between mice was very high (Fig. 4G). IL-2 is mostly produced by CD4^+^ T cells, reduced IL-2 abundance in the supernatant of total splenocytes could thus result from enhanced consumption by actively growing CD8^+^ T cells. The analysis of purified naïve CD4^+^ T cells confirmed a tendency toward diminished IL-2 (Fig. 4H) and enhanced IFN γ (Fig. 4G) production in CD4-Cre-SNX27^fl/+^ CD4^+^ cells compared to controls. These findings indicate that SNX27 partial deficiency moderately affects the capacity of mouse T cells to secrete cytokines under our ex vivo stimulation conditions.

### SNX27 partial deficiency in T cells impairs CD4^+^ T cell expansion upon activation

**Fig. 5:**
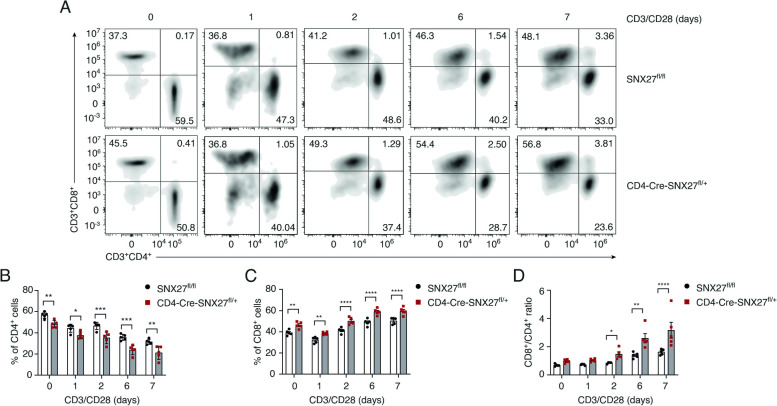
CD4-Cre-SNX27^fl/+^ mice show altered CD4^+^ and CD8^+^ T cell expansion upon activation. **A-D** Splenocytes from SNX27^fl/fl^ or CD4-Cre-SNX27^fl/+^ mice were stimulated 1, 2, 6 or 7 days with plate-bound anti-CD3 (2.5 µg/ml) and soluble anti-CD28 (1.25 µg/ml). Collected cells were stained for flow cytometry analysis with LIVE/DEAD violet dead cell stain followed by incubation with mouse-directed anti-CD19, anti-CD3, anti-CD4 and anti-CD8 antibodies (gating strategy is shown in Suppl Fig. 4). **A** Representative density plots from one series of experiments showing the percentage of CD4^+^ and CD8^+^ T cells (CD19^−^ CD3^+^). **B** Percentage of CD4^+^ cells (CD19^−^ CD3^+^ CD4^+^), (**C**) CD8^+^ cells (CD19^−^ CD3^+^ CD8^+^), and (**D**) the ratio between CD8^+^/CD4^+^ T cells is shown. Data shown as mean ± SEM; *p < 0.05; ****p < 0.0001; two-way ANOVA/Bonferroni post-test was used for multiple comparisons; *n* = 5 mice. *SNX* Sorting nexin, *SEM* Standard error of the mean

The previous analysis indicates that enhanced proliferation in the absence of augmented IL-2 production could favor expansion of the CD8 pool. Next, we examined the consequences of partial SNX27 deletion during ex vivo T cell activation on the simultaneous expansion of CD4^+^ and CD8^+^ T cells. Splenocytes from SNX27^fl/fl^ and CD4-Cre-SNX27^fl/+^ mice were stimulated with plate-bound anti-CD3 in combination with soluble anti-CD28 and the percentage of CD4^+^ and CD8^+^ T cells was determined by flow cytometry at the indicated times. Both genotypes showed a similar trait with a reduction in the CD4^+^ pool and concomitant massive expansion of the CD8^+^ T cells, as it is generally observed in the absence of exogenous addition of IL-2 (Fig. 5A, B, C). However, the reduction in CD4^+^ cells and the increment of CD8^+^ cells was greater in the CD4-Cre-SNX27^fl/+^ mice compared to the SNX27^fl/fl^ mice (Fig. 5B, C). As a result, the CD8^+^/CD4^+^ ratio was significantly increased at the end of the experiment (Fig. 5D).

### CTL from CD4-Cre-SNX27^fl/+^ mice show enhanced secretion of cytotoxic molecules and inflammatory cytokines

**Fig. 6:**
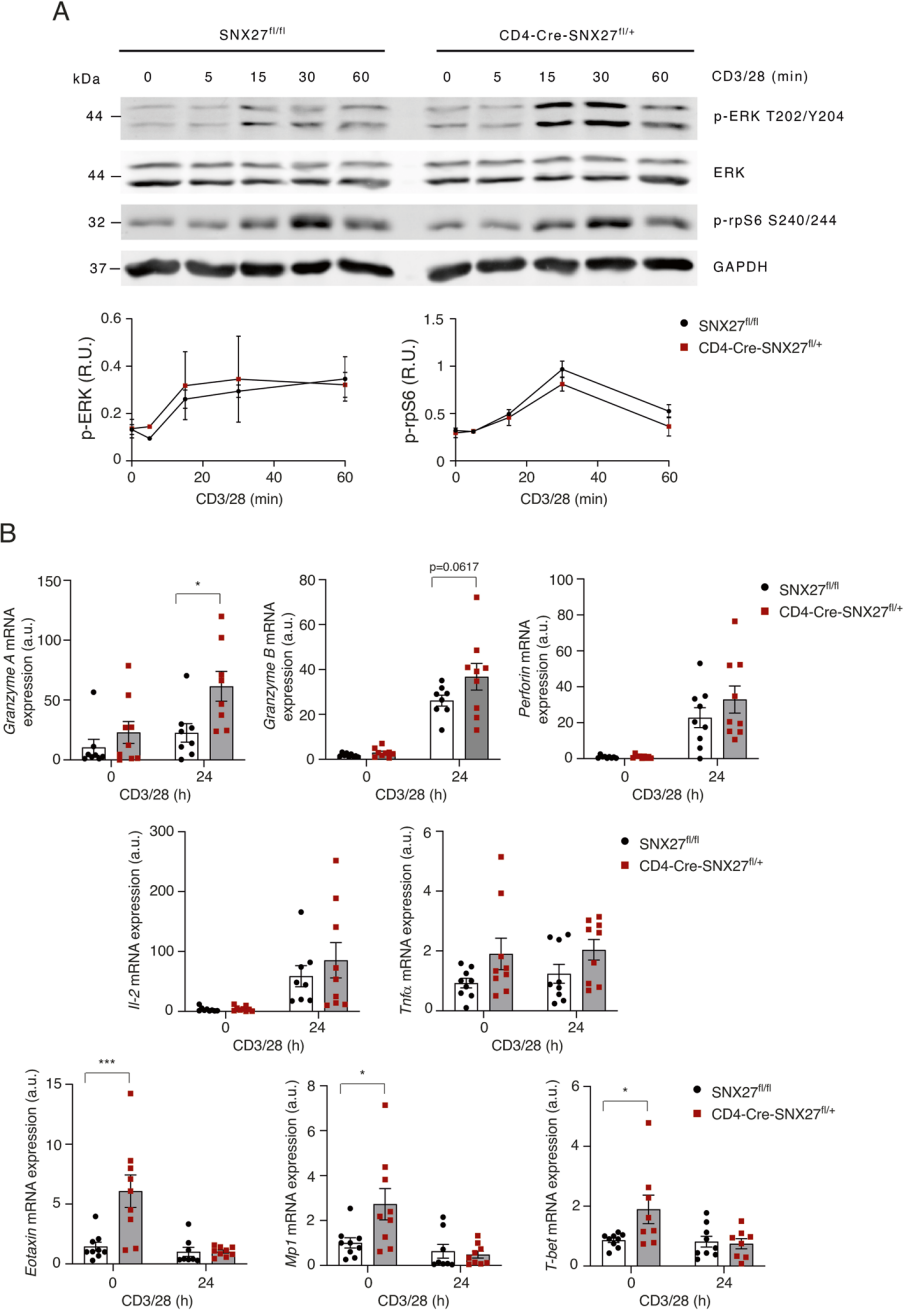
CTL heterozygous for SNX27 show enhanced expression of inflammatory and cytotoxic molecules. **A** Ex vivo-differentiated CTL from SNX27^fl/fl^ or CD4-Cre-SNX27^fl/+^ mice were stimulated with soluble anti-mouse CD3 and CD28 (1 μg/ml) for the indicated times. Cell lysates were analyzed by Western blot using the depicted antibodies. GAPDH was used as a loading control. A representative blot and the quantification of p-ERK and p-rpS6 levels are shown (*n* = 3 mice). Values were normalized against GAPDH. Data are shown as mean ± SEM; two-way ANOVA with Bonferroni post-test was used for multiple comparisons; ns *p* > 0.05. **B** Ex vivo*-*differentiated CTL from SNX27^fl/fl^ or CD4-Cre-SNX27^fl/+^ mice were stimulated with plate-bound anti-CD3 (2.5 μg/mL) and soluble anti-CD28 (1.25 μg /mL) for 24 h. Relative expression of the indicated genes was calculated by qPCR using de ΔΔCT method. β-actin was used as control. Data are shown as mean ± SEM; *p < 0.05. Two-way ANOVA with Bonferroni post-test was used for multiple comparisons. *n* = 9 mice. *SNX* Sorting nexin, *CTL* Cytotoxic lymphocytes, *SEM* Standard error of the mean

The expansion of naïve CD8^+^ cells triggers the onset of the cytotoxic program, which facilitates their differentiation into CTL, key players of the adaptive immune system in the clearance of infected cells [23]. To act against infection, activated CTL release cytotoxic granules that contain a pore-forming protein called perforin, as well as proteases termed granzymes, like granzyme A and B [24]. Given the higher expression of CD8^+^ cells as the result of SNX27 deficiency, we next investigated the consequences of SNX27 partial depletion on CTL differentiation and cytotoxic function. To obtain CTL, CD8^+^ T cells were stimulated with anti-CD3/CD28 and differentiated during 6 days with IL-2. Then, the ex vivo*-*differentiated CTL from SNX27^fl/fl^ and CD4-Cre-SNX27^fl/+^ mice were stimulated with soluble anti-mouse CD3 and CD28 for the indicated times and cell lysates were analyzed by western blot. Quantification of phosphorylation of ERK at its T202/Y204 residue and phosphorylation of mTORC-1 dependent S6 kinase (S6K) at its Ser240/244 residue showed similar ERK and slight decreased mTOR activation in CD4-Cre-SNX27^fl/+^ mice (Fig. 6A). This observation partially resembles the decreased S6K phosphorylation observed in SNX27-silenced Jurkat T cells and splenocytes derived from SNX27 *knockout* (KO) mice upon CD3/CD28 and Concanavalin A stimulation [17]. Next, we studied the mRNA expression of lytic enzymes and pro-inflammatory cytokines by qPCR in ex vivo-differentiated CTL in resting conditions and 24 h after stimulation with CD3/CD28. CD4-Cre-SNX27^fl/+^ CTL showed upregulated expression of *Granzyme A* compared to SNX27^fl/fl^ ones (Fig. 6B). Although mRNA levels of G*ranzyme B*, *Perforin* and *Il-2* mRNA also appeared to be increased, these changes were not statistically significant (Fig. 6B). Analysis of *Tnfα* expression revealed a significant increment in the transcription of this cytokine before stimulation in CD4-Cre-SNX27^fl/+^ CTL compared to SNX27^fl/fl^ CTL (Fig. 6B). Studies in individuals with DS demonstrate enhanced expression of *Eotaxin*, *Mp1* and *T-bet* by CD8 naïve T cells after stimulation with CD3/CD28 [18, 19], important mediators in inflammation and immunity against pathogens. Analysis of the expression of these molecules showed higher mRNA expression in basal conditions in SNX27 heterozygous CTL (Fig. 6B). Next, we sought out to determine whether the increased translation of lytic enzymes observed in CD4-Cre-SNX27^fl/+^ CTL resulted in an altered cytotoxicity. To do so SNX27^fl/fl^ or CD4-Cre-SNX27^fl/+^ CTL were stimulated with plate-bound anti-CD3 and soluble CD28 for 3 h and CD107a surface expression was assessed by flow cytometry, which did not show significant differences between both mice genotypes (Suppl Fig. 7A, B). Analysis of the killing capacity of SNX27^fl/fl^ and CD4-Cre-SNX27^fl/+^ CTL using a lactate dehydrogenase-release colorimetric assay was also comparable between both mice genotypes (Suppl Fig. 7C). These results demonstrate that partial SNX27 deficiency alters the CTL cytotoxic program with enhanced transcription of cytolytic and pro-inflammatory genes but does not affect their killing capacity under our ex vivo experimental setup.

### SNX27 partial deficiency impairs MTOC polarization in naïve CD4^+^ T cells but not in CTL

**Fig. 7:**
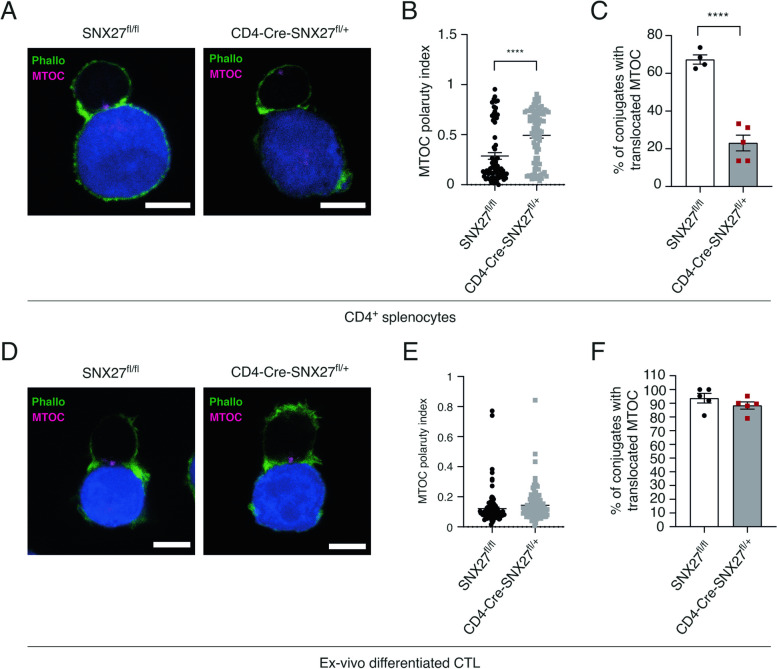
SNX27 partial deletion impairs MTOC polarization in CD4^+^ T cells but not in CTL. **A** Isolated naïve CD4^+^ T cells from SNX27^fl/fl^ and CD4-Cre-SNX27^fl/+^ mice were set to interact with CMAC-labeled (blue), anti-CD3-coated (2.5 µg /ml) P815 cells for 15 min. Cells were then fixed with 2% PFA and co-stained for F-actin (phalloidin-A488) and MTOC (anti-pericentrin) followed by anti-rabbit IgG-Cy5. Confocal optical sections were acquired at 0.2 µm intervals. Representative confocal sections are depicted. Scale bar = 5 µm. Quantification of (**B**) MTOC polarity index and (**C**) percentage of cell conjugates with a polarized MTOC is shown. The polarity index is calculated as the ratio between the distance from the MTOC to the IS and the distance from the IS to the distal pole of the cell. When the relative distance was < 0.25 µm, MTOC was considered to be polarized. Ratio values are displayed as dot plots, with each dot representing an individual cell. Data shown as mean ± SEM; ****p < 0.0001; unpaired *t* test; *n* = 71 SNX27^fl/fl^ cells, 95 CD4-Cre-SNX27^fl/+^ cells in (**B**), and *n* = 5 mice in (**C**). **D** Ex vivo-differentiated CTL from SNX27^fl/fl^ and CD4-Cre-SNX27^fl/+^ mice were incubated with CMAC-labeled (blue), anti-CD3 coated (2.5 µg /ml) P815 target cells during 15 min and stained as described in (**A**). Quantification of (**E**) MTOC polarity index and (**F**) percentage of cell conjugates with a polarized MTOC is shown. Data shown as mean ± SEM; unpaired *t*-test; *n* = 107 SNX27^fl/fl^ cells, 104 CD4-Cre-SNX27^fl/+^ cells in (**E**), and *n* = 5 mice in (**F**). *SNX* Sorting nexin, *CTL* Cytotoxic T lymphocytes, *SEM* Standard error of the mean

The analysis of CD4-Cre-SNX27^fl/+^ mice reveals a loss of peripheral CD4^+^ T cell pool that alters the CD4^+^/CD8^+^ ratio. Early studies demonstrated that long-term maintenance of CD4^+^ T cells in the periphery requires continuous contact with MHC-II molecules that provide survival signals [25]. Studies in human T cell lines demonstrated that SNX27 silencing impairs microtubule organization at the IS [21]. Thus, we reasoned that polarization defects in naïve CD4^+^ T cells could be related to the observed alterations on this population. To mimic the interaction between T cells and antigen-presenting cells, isolated naïve CD4^+^ T cells from SNX27^fl/fl^ or CD4-Cre-SNX27^fl/+^ mice were incubated 15 min with anti-CD3-coated P815 target cells and stained with antibodies that recognize pericentrin and phalloidin, for visualization of the microtubule organizing center (MTOC) and F-actin respectively. Formation of an actin ring at the cell–cell interface was indicative of the establishment of an IS (Fig. 7A). When in contact with target cells, purified CD4^+^ T cells from SNX27^fl/fl^ mice showed MTOC polarization to the contact area (Fig. 7A**, **left panel). On the contrary, CD4-Cre-SNX27^fl/+^ CD4^+^ T cells failed to polarize the MTOC (Fig. 7A**, **right panel). Analysis of MTOC polarity indexes confirmed significantly greater values in CD4-Cre-SNX27^fl/+^ CD4^+^ T cells (Fig. 7B). While an average of 67% of control CD4^+^ T cells bound to P815 displayed a translocated MTOC, only 23% of CD4-Cre-SNX27^fl/+^ CD4^+^ T cells did (Fig. 7C).

The structure of the cytotoxic synapse between a CTL and its target cell resembles the one formed between a CD4^+^ and an antigen presenting cell (APC) [26]. Efficient TCR stimulation leads to targeted polarization towards the cell–cell junction of cytotoxic granules, which are later released into the synaptic cleft [27, 28]. We next investigated the organization of cytotoxic IS by incubating ex vivo*-*differentiated CTL and anti-CD3-coated P815 target cells (Fig. 7D). In contrast to that observed for naïve CD4^+^ T cells, CTL derived from SNX27^fl/fl^ mice or CD4-Cre-SNX27^fl/+^ were equally able to position the MTOC at the cell–cell interface (Fig. 7D, E, F). These data confirm the relevance of SNX27 for MTOC positioning in naïve CD4^+^ T cells and demonstrate that this requirement is not necessary in CTL.

## Discussion

Our previous studies with T cell lines identified SNX27 as a critical hub that links microtubule organization and cargo delivery to the IS and suggested that defects in this protein could have important consequences in T cell-regulated responses. To explore the contribution of SNX27 in primary T cells, we developed a mouse model in which a copy of SNX27 was selectively deleted in the T cell lineage. CD4-Cre-SNX27^fl/+^ mice behave as the WT in terms of weight and development of the lymphoid organs. Of note, these mice display a decreased CD4^+^/CD8^+^ T cells ratio in circulating blood and hematopoietic organs which evidenced a contribution of SNX27 in T cell homeostasis. The reduced frequencies of circulating CD4^+^ T cells and B cells, and the inverted CD4^+^/CD8^+^ ratios determined in the blood and lymphoid organs of CD4-Cre-SNX27^fl/+^ mice compared to SNX27^fl/fl^ ones, are reminiscent of age-associated changes in mice and human [29]. Decreased T cell numbers have been reported in human aging and are considered to have an important contribution to age-related immune defects. A relative decline of naïve CD4^+^ T cells in blood, spleen, and thymus has also been observed in telomerase deficient (mTerc^−/−^) mice [30], which also correlates well with age-associated changes found within the human immune system.

An imbalance in the T cell subset is a well-characterized abnormality in T21. Since early age, individuals with DS show a reduction in the number of circulating CD4^+^ T cells compared to age-matched controls [18, 19, 31–33]. People with DS also exhibit a reduced number of total circulating B cells and present important alterations at all stages of their differentiation [18, 32–34]. These defects could be B cell intrinsic, due to disturbances in T help or both. Aging-associated decrease in naïve T cells has been linked both to the reduction in thymic output due to thymic involution, as well as to insufficient homeostatic maintenance of naïve T cells in the periphery [35]. Whether the immune system in T21 is intrinsically deficient from the beginning or is the result of a generalized process of precocious aging is a matter of debate. Some authors relate the decreased presence of naïve B and T cells to deficient production from birth [36] whereas others suggest early senescence of the immune system [12]. Here, we did not find differences in thymus size indicative of thymic involution. Similarly, studies with mTerc^−/−^ mice [30], did not report histological differences in the thymus regarding organ size, architecture, or cellularity.

SNX27 partial deficiency did not alter the expression of naïve, memory and effector T cell pools. Nevertheless, we demonstrate enhanced proliferation of CD4-Cre-SNX27^fl/+^ CD4^+^ and CD8^+^ naïve T cells in response to TCR co-stimulation, which was not due to enhanced IL-2 production. Indeed, ELISA assessment of IL-2 production by splenocytes derived from CD4-Cre-SNX27^fl/+^ mice showed decreased IL-2 secretion compared to SNX27^fl/fl^ ones, in agreement with data previously reported for splenocytes derived from SNX27 KO mice [17]. Defects in IL-2 secretion linked to enhanced proliferation agree well with the observed defects in MTOC polarization during IS formation. Asymmetric inheritance of cellular content along division is important to sustain cellular viability and fitness. In T lymphocytes, asymmetric cell division represents an important mechanism to generate diversity, particularly in the formation of memory pools [37]. Recent studies suggest that aging leads to an overall decline in the ability of CD8^+^ T cells to undergo asymmetric cell division [38]. Our studies suggest that defects in MTOC polarization, as the result of SNX27 downregulation, could limit asymmetric division and promote age-related dysfunctions of the CD4^+^ T cell pool.

In contrast to that observed for naïve CD4^+^ T cells, analysis of CTL in conjugation with anti-CD3-coated P815 target cells suggests that SNX27 is not required for MTOC translocation to the cytotoxic IS. Proper polarization of MTOC in both SNX27^fl/fl^ and CD4-Cre-SNX27^fl/+^ derived CTL correlates with the absence of differences regarding their in vitro killing capacity. IL-2-dependent expansion of CD8^+^ T cells triggers the differentiation of effector CTL, stimulating the production of effector molecules perforin and granzymes [39]. qPCR analysis in CD4-Cre-SNX27^fl/+^ CTL revealed increased transcription of lytic enzymes upon activation. Interestingly, differentiated CTL expressed molecules characteristic of inflammation like eotaxin, a chemokine associated with aging and impaired cognitive function [40].

In summary, aging of the T cell compartment is the result of a complex combination of effects in immune cells, the microenvironment of lymphoid organs, and the presence of circulating factors [14]. A better characterization of true age-related changes requires examination of isolated populations. The findings made in this study have to be considered in light of some limitations, as only SNX27 deficiency in T cells was investigated in this study. Furthermore, the effector function of CD4^+^ T cells and CTL were only assessed in vitro and naïve CD8^ +^ T cells were not examined in detail. Therefore, further research on CD4-Cre-SNX27^fl/+^ mice as well as in vivo models will likely provide a more profound understanding of the effect of SNX27 deficiency on both CD4^+^ as well as CD8^ +^ T cells.

Still, the presented data strongly indicate that part of the changes observed in T cells of aged and individuals with DS could be linked to SNX27-associated mechanisms, which may be investigated in further studies. In addition to mice lacking SNX27 in the T cell compartment, future research employing mice with a deficiency of the protein in myeloid compartments might provide even more insight into the cellular roles of SNX27 in the control of immune responses.

## Conclusions

Here we demonstrate that partial deletion of SNX27 alters the distribution of circulating immune populations, enhances expression of cytotoxic and pro-inflammatory molecules, and impairs MTOC polarization during IS formation in CD4^+^ splenocytes. In conclusion, we provide innovative data on the role of SNX27 in T lymphocytes and demonstrate that the phenotype observed in CD4-Cre-SNX27^fl/+^ mice shows striking similarities not only to that observed in individuals with DS, but also in aging regarding the naïve T cell pool in steady-state as well as decreased IL-2 secretion, which contrasts with enhanced mRNA expression of well-characterized inflammatory cytokines and lytic enzymes. The data presented in this study highlights SNX27 as a key molecule in the regulation of immune responses and suggest that its deficiency can contribute to aging and to the immune dysregulation observed in T21.

### Materials and methods

#### Reagents and antibodies

Reagents used were: poly-L-lysine, bovine serum albumin (BSA), DAPI, orthovanadate (Na_3_VO_4_), phenylmethylsulphonyl fluoride (PMSF), sodium fluoride (NaF), leupeptin, aprotinin, Nonidet P-40 (NP40), β-glycerophosphate, phorbol 12-myristate 13-acetate (PMA), β-mercaptoethanol (all purchased from Sigma-Aldrich), paraformaldehyde (PFA) (Ted Pella), RPMI-1640, DMEM, L-glutamine penicillin/streptomycin, non-essential amino acids, sodium pyruvate, HEPES (all from Biowest), ionomycin, Tween-20, Triton X-100 (all purchased from Calbiochem), fetal bovine serum (FBS), TriZol, Pierce 660 nm Protein Assay Kit, Cell Tracker Blue 7-amino-4-chloromethylcoumarin (CMAC), CFSE (CellTrace violet), LIVE/DEAD violet dead cell stain (all from Thermo Fisher Scientific), H_2_SO_4_ (Merck), and ProLong Gold from Invitrogen.

For antibody-based stimulation, we used hamster anti-mouse CD3 (clone 145-2C11) and CD28 (clone 37.51) monoclonal antibodies (553,058, 553,295 BD Biosciences). For immunofluorescence staining we used pericentrin (ab4448, Abcam), Phalloidin-AlexaFluor 488 (A12379, Thermo Fisher), CD4-APC (17–0042-81, Invitrogen) and anti-rabbit IgG-Cy5 (111–176-046, Jackson ImmunoResearch). For flow cytometry we used: anti-mouse B220-PECy7 (103,222, Biolegend), anti-mouse CD3-FITC (731,979, Beckman Coulter), anti-mouse CD3-APC-eFluor780 (47–0032-82, Thermo Fisher Scientific), anti-mouse CD3-PECF594 (562,332, Bioscience), anti-mouse CD4-PECy7 (100,422, Biolegend), anti-mouse CD4-PerCPCy5.5 (100,434, Biolegend), anti-mouse CD8-APC (100,712, Biolegend), anti-mouse CD8-Bviolet605 (100,744, Biolegend), anti-mouse CD8-Efluor450 (48–0081-82 Thermo Fisher Scientific), anti-mouse CD8-PE (733,264, Beckman Coulter), anti-mouse CD11b-PECy7 (101,216, Biolegend), anti-mouse CD19-PE (1575-09L, Southern biotech), anti-mouse CD19-AF700 (56–0193-82, Thermo Fisher Scientific), anti-mouse CD44-APC (103,012, Biolegend), anti-mouse CD44-PE (1500-09L, Southern biotech), anti-mouse CD45-APC (17–0451-82, Thermo Fisher), anti-mouse CD62L-FITC (553,744, BD pharmingen), anti-mouse Ly6C-FITC (128,005, Biolegend), anti-mouse Ly6G-PE (551,461, BD pharmingen) and anti-mouse Ter119-PECy7 (116,221, Biolegend). For western blot, we used anti-SNX27 (mouse) (provided by Dr. Wanjin Hong), anti-ERK1/2, -pERK1/2 (T202/Y204),—P70 S6 Kinase, -pP70 S6 Kinase (S240/244) (4696, 9101, 2708, 2215; Cell Signaling), and anti-α-tubulin (T9026; Sigma Aldrich). The following secondary antibodies for western blot were used: anti-rabbit IgG Dylight 800 (SA5-35,571; Thermo Scientific), AlexaFluor 680-anti-mouse IgG (A-21057; Life Technologies), anti-rabbit IgG StarBrightTM Blue 700, IgG StarBrightTM Blue 520, anti-mouse IgG StarBrightTM Blue 700, and IgG StarBrightTM Blue 520 (12,004,161, 12,005,869, 12,004,158, 12,005,866; BioRad).

### Mice

CD4^+^-specific-SNX27 deficient mice were generated by backcrossing SNX27-floxed mice (SNX27^flox/flox^), a kind gift from Dr. Paul Slesinger (University of California, USA), with CD4-Cre transgenic mice. CD4^+^-Cre-SNX27 heterozygotes mice (CD4-Cre-SNX27^fl/+^) were kept in a C57BL/6 genetic background. SNX27^flox/flox^ mice were used as controls. Validation of SNX27-specific depletion was performed by PCR using the following primers: 5′-ACCATAGGGTTGATGAGATGGCCAA-3′ and 3′ ACAGCATGACTTGGCTGACATACGTG-5´. Mice were bred in pathogen-free conditions in the animal facility of the National Centre for Biotechnology and handled according to European and Spanish regulations (2010/63/UE; RD 53/2013).

### Harvesting of hematopoietic organs, cell isolation and culture

SNX27^fl/fl^ and CD4-Cre-SNX27^fl/+^ male and female mice were sacrificed at 9–12 weeks of age. Spleen, thymus, and bone marrow were harvested. Spleen and thymus weight and size were determined, and cells from these organs were extracted and total cellularity was analyzed using a cell counter. Bone marrow cells were isolated from the hind legs of the mice (femur and tibia bones) by flushing 10 ml of RPMI medium through the bone using a syringe with 25-gauge needle, and naïve, memory, and effector cell subsets were analyzed.

For lymphocyte isolation, splenocytes were mechanically disaggregated in phosphate-buffered saline (PBS), cells were collected using a 40 µm cells strainer (BD Biosciences) and treated with erythrocyte lysis buffer. For CD4^+^ T cells isolation MojoSort Mouse CD4 T Cell Isolation Kit was used, and cells were cultured in mouse medium: RPMI supplemented with 10% FBS, 2 mM L-glutamine, 1 mM sodium pyruvate, 1% nonessential amino acids, 50 µM β-mercaptoethanol, 10 mM HEPES, and penicillin–streptomycin (100 U/ml), and further used in the experiments. To analyze CTL in CD4-Cre-SNX27^fl/+^ mice, mouse CD8^+^ cells were purified from isolated splenocytes using the MojoSort Mouse CD8 T cell Isolation Kit. Then CD8^+^ cells were cultured for 2 days in plates coated with anti-mouse CD3 (2.5 μg/ml), soluble anti-mouse CD28 (1.25 μg/ml) and IL-2 (100 U/ml). Then fresh IL-2 (100 U/ml) was added every two days, and experiments were performed after 6 days.

Individual mice were analyzed separately, and no significant gender effect was observed in any of the experiments performed. Data from male and female mice were pooled for each experimental condition.

The mouse mastocytoma cell line P815, kindly donated by Dr. Andrés Alcover, was used as tumor target cell.

### Cell stimulation

For stimulation of primary T cells, cells were incubated in complete mouse medium (1 × 10^6^ cells/ml) with plate-bound anti-mouse CD3 (2.5 µg/ml) supplemented with anti-mouse CD28 (1.25 µg/ml).

For conjugate formation with CD4^+^ T cells and CTL, the mouse mastocytoma cell line P815 was used to generate stimulatory cells. P815 were stained in complete DMEM medium with 10 μM CMAC (1 h, 37 °C, darkness). Labeled P815 were washed twice in PBS and were coated with anti-mouse CD3 antibody (2.5 µg/ml) (45 min, 37 °C). Coated P815 were then added at 1:1 ratio on top of primary T cells previously plated on poly-L-lysine-coated coverslips. Cells were incubated for the indicated times (37 °C, 5% CO2), after which immunofluorescence was performed.

For analysis of CTL activation, cells (1 × 10^6^ cells/ml) were stimulated with soluble anti-mouse CD3 and CD28 (1 μg/ml). After the incubation period, cells were collected and analyzed by western blot.

### Hematological analysis

Mouse blood was collected from the heart using a syringe with 21-gauge needle and introduced in polypropylene tubes containing ethylene diamine tetra acetic acid (EDTA). Aliquots of whole blood were then analyzed using an Abacus Junior Vet, automated hematological analyzer (CVM SL.). The parameters analyzed were: white blood cells (WBC), lymphocytes, monocytes and granulocytes count and percentage, red blood cells (RBC) counts, hemoglobin concentration, hematocrit (HCT) and platelets count.

### Flow cytometry analysis

For analysis of circulating immune populations in blood samples aliquots of whole blood were incubated with a mix of LIVE/DEAD violet fixable cell stain and mouse-directed anti-CD45-APC, anti-CD3-APC-eFluor780, anti-B220-PECy7, anti-CD8-Bviolet605, anti-CD4-PerCPCy5.5, antiLy6C-FITC and anti-Ly6G-PE antibodies (20 min, RT). Versalyte solution was then added to lyse red cells (10 min, RT, darkness) and the percentage of cells of the following populations was analyzed by flow cytometry: B cells (CD45^+^ CD3^−^ B220^+^), T cells (CD45^+^ CD3^+^ B220^−^), CD4^+^ and CD8^+^ (CD45^+^ B220^−^ CD3^+^), monocytes (CD45^+^ CD3^−^ B220^−^ Ly6C^+^), inflammatory monocytes (CD45^+^ CD3^−^ B220^−^ Ly6C^+^ high) and granulocytes (CD45^+^ CD3^−^ B220^−^ Ly6G^+^). Gating strategy is shown in Suppl Fig. 3.

For analysis of splenocytes, bone marrow cells and thymocytes cells (gating strategies are depicted in Suppl Figs. 1, 4 and 5) were collected in ice-cold PBS and cell surface proteins stained with saturating concentrations of the indicated fluorophore-conjugated primary antibodies in PBS staining buffer (1% FBS, 0.5% BSA, 0.01% sodium azide/PBS) 30 min at 4 ºC. The purity of the isolated CD4^+^ or CD8^+^ T cells was analyzed by flow cytometry by staining with anti-mouse CD3-FITC, anti-mouse CD19-PE, anti-mouse CD8-APC and anti-mouse CD4-PECy7 antibodies. Cells were washed and fixed using 1% PFA and maintained at 4 ºC for flow cytometry using Cytomix FC500, Galios Cytometer or Cytoflex (Beckman Coulter). Cells and singlets were gated using forward and side scatter parameters. LIVE/DEAD violet dead fixable cell strain was used when compatible with the fluorophore panel. Data were analyzed using FlowJo software (TreeStar).

### Proliferation assay

Flat-bottom 96 well-plates were prepared with or without anti-mouse CD3 coating (2.5 μg/mL). Splenocytes were stained in warm PBS with 0.5 μM CFSE (15 min, 37 °C, darkness). Unbound dye was quenched by adding five times the staining volume of FBS-containing medium (5 min, 37 °C). Cells were then washed twice in FBS-containing medium. 100,000 splenocytes were seeded per well and were additionally incubated with soluble anti-mouse CD28 (1.25 μg/mL). Afterwards, cells were washed twice in cold PBS and stained with LIVE/DEAD violet fixable stain, followed by staining with mouse-directed anti-CD4-PECy7, anti-CD8-APC and anti-CD19-PE antibodies. CD4^+^ or CD8^+^ T cells were gated on live, CD19^−^ splenocytes and dye dilution analysis of T cell division was carried out by flow cytometry. Gating strategy is shown in Suppl Fig. 6.

### Enzyme-linked immunosorbent spot assay (ELISA)

To assess IL-2 and IFNγ cytokine production, total splenocytes or isolated naïve CD4^+^ T cells from SNX27^fl/fl^ or CD4-Cre-SNX27^fl/+^ mice were stimulated 48 h with anti-CD3 (2.5 µg/ml) and soluble anti-CD28 (1.25 µg/ml). Afterwards, the concentration of the depicted cytokines in the supernatant was assessed by sandwich ELISA (Biolegend) according to the manufacturer´s instructions.

### Western blot

For western blot analysis, cells were washed in ice-cold PBS and lysed in NP40 lysis buffer (10 mM HEPES pH 7.5, 15 mM KCl, 1 mM EGTA, 1 mM EDTA, 1% NP40, 10% glycerol) containing protease and phosphatase inhibitors (2 nM NaF, 40 mM β-glycerophosphate, 20 μM leupeptin, 1.5 μM aprotinin, 1 mM PMSF and 1 mM Na_3_VO_4_). Lysates were clarified and quantified using the Pierce 660 nm Protein Assay. A similar amount of protein per sample was analyzed by SDS-PAGE, transferred to nitrocellulose membranes (BioRad) and incubated with each of the indicated primary antibodies. Detection of fluorescent-conjugated secondary antibodies was carried out on a ChemiDocTM MP Imaging System (BioRad).

### Gene expression by real-time qPCR

Total RNA was isolated from cell cultures (2–5 × 10^6^ cells) using Trizol reagent. Complementary DNA (cDNA) was synthesized from isolated RNA (2 μg) after retrotranscription with Randrom Primers (Applied Biosystems) and Multiscribe Reverse Transcriptase (Applied Biosystems). qPCR reactions were performed with Evagreen qPCR mix plus (Cultek molecular Bioline) in 8 μl final volume. PCR conditions were as follows: 95ºC (10 min), 40 cycles at 95ºC (15 s) and 60ºC (1 min), continued by 95ºC (15 s), 60ºC (1 min) and 95ºC (15 s). Reactions were run in triplicate with the Applied Biosystems QuantStudio 5 System. β-actin was used as control because this gene was stably expressed in all cases. All samples were subjected to a melting curve analysis to verify the single amplification product. Relative expression of each gene was calculated using the ΔΔCt method. qPCR reactions were run in triplicates on each sample, and three biological replicates were performed. Primer sets used were:

- Mouse *Granzyme A*:
- Forward – TTTCATCCTGTAATTGGACTAA
  Reverse – GCATCTCCACACTTCTC.

- Mouse *Granzyme* B:
- Forward – CCTCCTGCTACTGCTGAC
  Reverse – GTCAGCACAAAGTCCTCTC

- Mouse *Perforin*:
- Forward - CGTCTTGGTGGGACTTCAG
  Reverse – GCATTCTGACCGAGTGGCAG

- Mouse *Il-2*:
- Forward - TTCAATTGGAAGATGCTGAGA
- Reverse - ATCATCGAATTGGCACTCAA

- Mouse *Tnfα*:
- Forward - CTGTAGCCACGTCGTAGC
- Reverse - TTGAGATCCATGCCGTTG

- Mouse *Eotaxin*:
- Forward - GCAGGAAGTTGGGATGGA
- Reverse - AGAGCTCCACAGCGCTTCT

- Mouse *Mp1*:
- Forward - CCAGTCCCTGTTCTTGACCA
  Reverse - AAGTCCAATTGTCACTGCATCTC

- Mouse *T-bet*:
- Forward - GGTGTCTGGGAAGCTGAGAG
  Reverse - GAAGGACAGGAATGGGAACA

### Degranulation assay

Differentiated mouse CTL were stimulated for the indicated time points with coated anti-mouse CD3 (5 μg/ml) and soluble anti-mouse CD28 (2.5 μg/ml) in the presence of CD107a-PE (LAMP1) (2.5 μg/ml). At each time point, cells were harvested in cold PBS and incubated on ice. At the end of the time course, cells were labeled with LIVE/DEAD violet dead cell stain followed by anti-mouse CD8-APC staining. Events were acquired on the flow cytometer. Singlets were gated for live cells, in which surface marker expression was analyzed.

### Cytotoxicity assay

Cytotoxicity was assessed using the Cytotox96 Non-Radioactive Cytotoxic Assay. Briefly, differentiated mouse CTL were resuspended in RPMI supplemented with 3% FBS, 2 mM L-glutamine and 10 mM HEPES. CTL were then co-cultured (4 h, 37 °C) at the indicated ratios with P815 target cells previously coated (45 min, 37 °C) with anti-mouse-CD3 (2.5 μg/ml). Lactate dehydrogenase released by dead target cell was measured at 490 nm using an absorbance microplate reader. The percentage of target cell lysis was calculated following manufacturer´s instructions.

### Immunostaining

For immunofluorescence labeling, cells were fixed with 2% PFA (15 min, RT). After washing twice with PBS, cells were blocked and permeabilized for 30 min at RT (1% BSA, 0.1% Triton X-100 in PBS). This buffer was also used throughout the procedure as staining buffer. Cells were incubated with the corresponding primary antibodies (1 h, RT), washed with PBS, and incubated with the corresponding fluorophore conjugated secondary antibodies (30 min, RT). Coverslips were washed twice in PBS and mounted on glass slides using ProLong Gold.

### Confocal microscopy and image processing

Confocal images were acquired using a Leica TCS SP8 confocal microscope equipped with a Plan-Apochromat HCX PL APO 63 × 1.4 NA oil immersion objective or using the Olympus Fluoview FV1000 confocal laser-scanning microscope equipped with a uPlan-Apochromat 60 × 1.4 NA oil objective lens. Images were collected with LAS X (Leica) or the FV10-ASW4.2 (Olympus) acquisition softwares. Sets to be compared were acquired using the same acquisition settings. To quantify protein, fluorescence images were processed using ImageJ software (NIH).

For CD4 localization assays, z-series optical Sects. (0.5 μm intervals) were collected and maximal intensity Z-projections of 4–6 contiguous optical planes, which included the three-dimensional fluorescence information, were stacked. The mean fluorescence intensity (MFI) was measured in the whole cell in basal conditions.

To measure the ability of the MTOC to translocate to the IS, cell z-stacks (0.2-µm intervals) were acquired, and maximum intensity projections were generated using the Fiji software [41]. The geometric center of MTOC (MTOC^C^) as well as the IS region were determined. The polarity index was computed by dividing the distance between MTOC^C^ to the IS (“a” distance) by the distance from the IS to the distal pole of the T cell (“b” distance). This allowed polarity indexes to be normalized to cell size and shape. We considered polarization to occur when the polarity index was < 0.25. Polarity index values are represented in graphs as dot plots, where each dot represents an individual cell.

### Statistical analysis

Statistical analysis was performed with GraphPad Prism 8 software, and samples were assumed to fit a normal distribution. Details about the data presentation, the experimental replication, and the statistical tests used are included in the individual figure legends. Briefly, an unpaired student’s *t*-test was used to analyze differences between two conditions. Two-way ANOVA with a Bonferroni post-test was applied for multiple comparisons. The level of statistical significance is represented by * *p* < 0.05; ** *p* < 0.01; *** *p* < 0.001; ****p < 0.0001. Data are shown as mean ± standard error of the mean (SEM) unless otherwise specified.

### Supplementary Information

**Additional file 1: Supplemental Figure 1. **Flow cytometry gating strategy for analysis of lymphocyte populations in thymus. Double positive (DP; CD8^+^CD4^+^) and single positive (SP; CD8^+^CD4^-^ or CD8^-^CD4^+^) thymocytes were identified.

**Additional file 2: Supplemental Figure 2.** Hematological parameters are not affected by SNX27 depletion in CD4-Cre-SNX27^fl/+^ mice. (A-K) Aliquots of whole blood from SNX27^fl/fl^and CD4-Cre-SNX27^fl/+^ mice were analyzed using an Abacus Junior Vet automated hematological analyzer to determine the following hematological parameters: (A) WBC count, (B, C) lymphocyte, (D, E) monocyte and (F, G) granulocyte count and percentage, (H) RBC count, (I) hemoglobin concentration, (J) HCT percentage and (K) platelets count. Data are shown as mean ± SEM; ns p>0.05; unpaired *t*-test (*n*=12 mice). SNX: Sorting nexin; WBC: White blood cells; RBC: Red blood cells; HCT: Hematocrit; SEM: Standard error of the mean.

**Additional file 3: Supplemental Figure 3. **Flow cytometry gating strategy for circulating immune cell populations study. B cells (CD45^+^ CD3^-^ B220^+^), T cells (CD45^+^ CD3^+^ B220^-^), CD4^+^, CD8^+^ (CD45^+^ B220^-^ CD3^+^), monocytes (CD45^+^ CD3^-^ B220^-^ Ly6C^+^), inflammatory monocytes (CD45^+^ CD3^-^ B220^-^ Ly6C^+^ high), and granulocytes (CD45^+^ CD3^-^ B220^-^ Ly6G^+^) were identified.

**Additional file 4: Supplemental Figure 4. **Flow cytometry gating strategy for analysis of CD4^+^ and CD8^+^ cells in bone marrow and spleen. Gating strategies used to identify CD4^+^ and CD8^+^ cells in bone marrow (A) and spleen (B).

**Additional file 5: Supplemental Figure 5.** Flow cytometry gating strategy for analysis of naïve, memory and effector T cell subsets. Gating strategies used to identify naïve, central memory and effector cells in blood (A), bone marrow (B), and spleen (C).

**Additional file 6: Supplemental Figure 6. **Flow cytometry gating strategy for analysis of CD4^+^ and CD8^+^ proliferation. CD4^+^ and CD8^+^ splenocytes were identified and the dilution of the CFSE marker was assessed.

**Additional file 7: Supplemental Figure 7.** Absence of SNX27 does not affect CTL LAMP-1 expression nor killing capacity. (A) Ex vivo-differentiated CTL from SNX27^fl/fl^or CD4‑Cre‑SNX27^fl/+^ mice were stimulated for 3 h with plate‑bound anti-CD3 (5 μg/ml) and soluble anti-CD28 (2.5 μg/ml) in the presence of CD107a-PE (LAMP1) (2.5 μg/ml). Afterwards, surface analysis of CD107a was analyzed by flow cytometry. A representative flow cytometry plot is shown. (B) GMFI of surface CD107a. Data are shown as mean ± SEM; ns p>0.05; two-way ANOVA with Bonferroni post-test was used for multiple comparisons; *n*=5 mice. (C) Ex vivo‑differentiated CTL from SNX27^fl/fl^or CD4‑Cre‑SNX27^fl/+^ mice were mixed with anti-CD3-coated P815 target cells (2.5 μg/ml) for 4 h at the depicted CTL/target cell ratios. Killing was examined by a colorimetric assay, as described in the methods section. A representative experiment is shown (n >3 mice). SNX: Sorting nexin; CTL: Cytotoxic T lymphocyte; GMFI: geometric mean fluorescence intensity; SEM: Standard error of the mean.

## Data Availability

Data and material presented in this study are available upon request.

## Abbreviations

APC: Antigen presenting cell
BSA: Bovine serum albumin
cDNA: Complementary DNA
CMAC: CellTracker Blue 7-amino-4-chloromethylcoumarin
CTL: Cytotoxic T cell
DP: Double positive
DS: Down Syndrome
EDTA: Ethylenedinitrilo tetraacetic acid
EGTA: Ethyleneglycol tetraacetic acid
FSC: Forward scatter
GMFI: Geometric mean fluorescence intensity
HCT: Hematocrit
IS: Immune synapse
KO: Knock out
MFI: Mean fluorescence intensity
MTOC: Microtubule organizing center
mTOR: Mammalian Target of Rapamycin
NA: Numerical aperture
NaF: Sodium fluoride
NFκB: Nuclear factor
NP40: Nonidet P-40
PBS: Phosphate buffer saline
PDZ: Postsynaptic density 95/disc large/zonula occludens 1
PFA: Paraformaldehyde
PMA: Phorbol-12-myristate 13-acetate
PMSF: Phenylmethylsulphonyl fluoride
qPCR: Quantitative polymerase chain reaction
RBC: Red blood cells
RT: Room temperature
SEM: Standard Error of the mean
SNX: Sorting Nexin
S6K: MTORC-1 dependent S6 kinase
Tcm: T central memory
Teff: T effector
TCR: T cell receptor
TNFα: Tumor necrosis factor α
T21: Trisomy 21
WBC: White blood cells
