# Supplementary figures and images for: Partial loss of Sorting Nexin 27 resembles age- and Down syndrome-associated T cell dysfunctions
Source: Immun Ageing. 2024 Jan 2;21:2. doi: 10.1186/s12979-023-00402-3 (PMC10759489; doi:10.1186/s12979-023-00402-3)

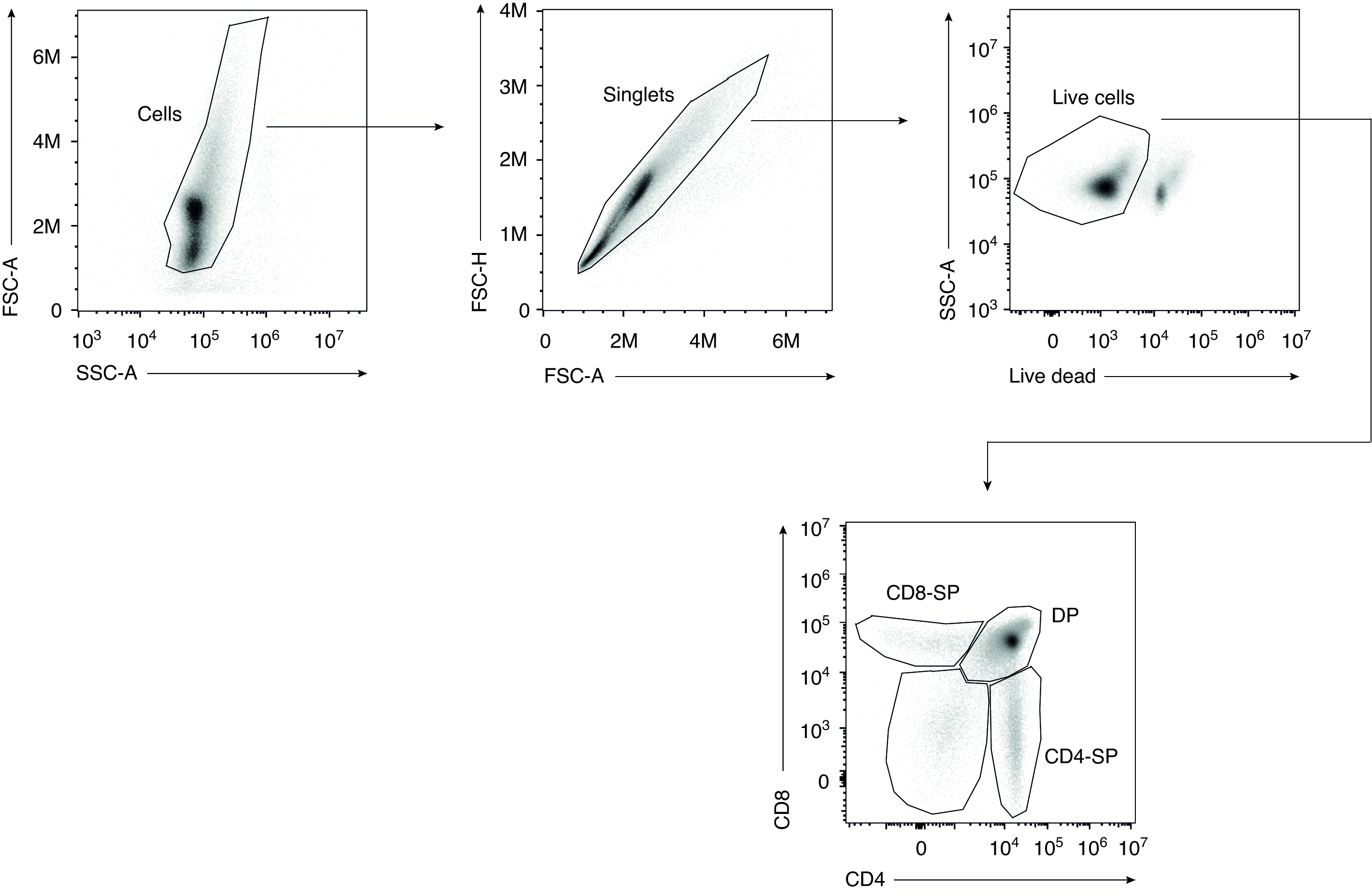

Supplement: Supplementary file 1 — Additional file 1: Supplemental Figure 1. Flow cytometry gating strategy for analysis of lymphocyte populations in thymus. Double positive (DP; CD8+CD4+) and single positive (SP; CD8+CD4- or CD8-CD4+) thymocytes were identified. [file 12979_2023_402_MOESM1_ESM.tif]

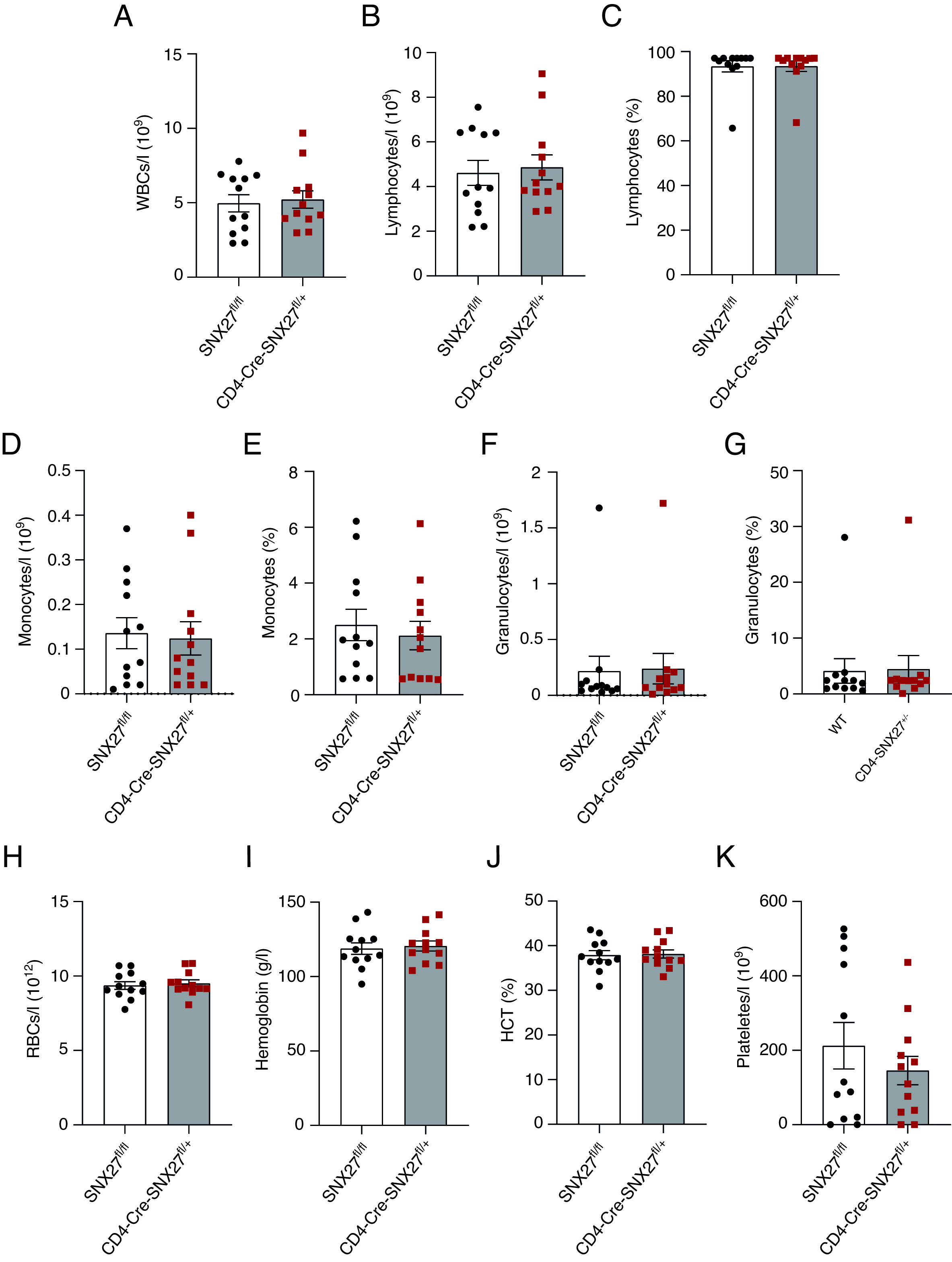

Supplement: Supplementary file 2 — Additional file 2: Supplemental Figure 2. Hematological parameters are not affected by SNX27 depletion in CD4-Cre-SNX27fl/+ mice. (A-K) Aliquots of whole blood from SNX27fl/fland CD4-Cre-SNX27fl/+ mice were analyzed using an Abacus Junior Vet automated hematological analyzer to determine the following hematological parameters: (A) WBC count, (B, C) lymphocyte, (D, E) monocyte and (F, G) granulocyte count and percentage, (H) RBC count, (I) hemoglobin concentration, (J) HCT percentage and (K) platelets count. Data are shown as mean ± SEM; ns p>0.05; unpaired t-test (n=12 mice). SNX: Sorting nexin; WBC: White blood cells; RBC: Red blood cells; HCT: Hematocrit; SEM: Standard error of the mean. [file 12979_2023_402_MOESM2_ESM.tif]

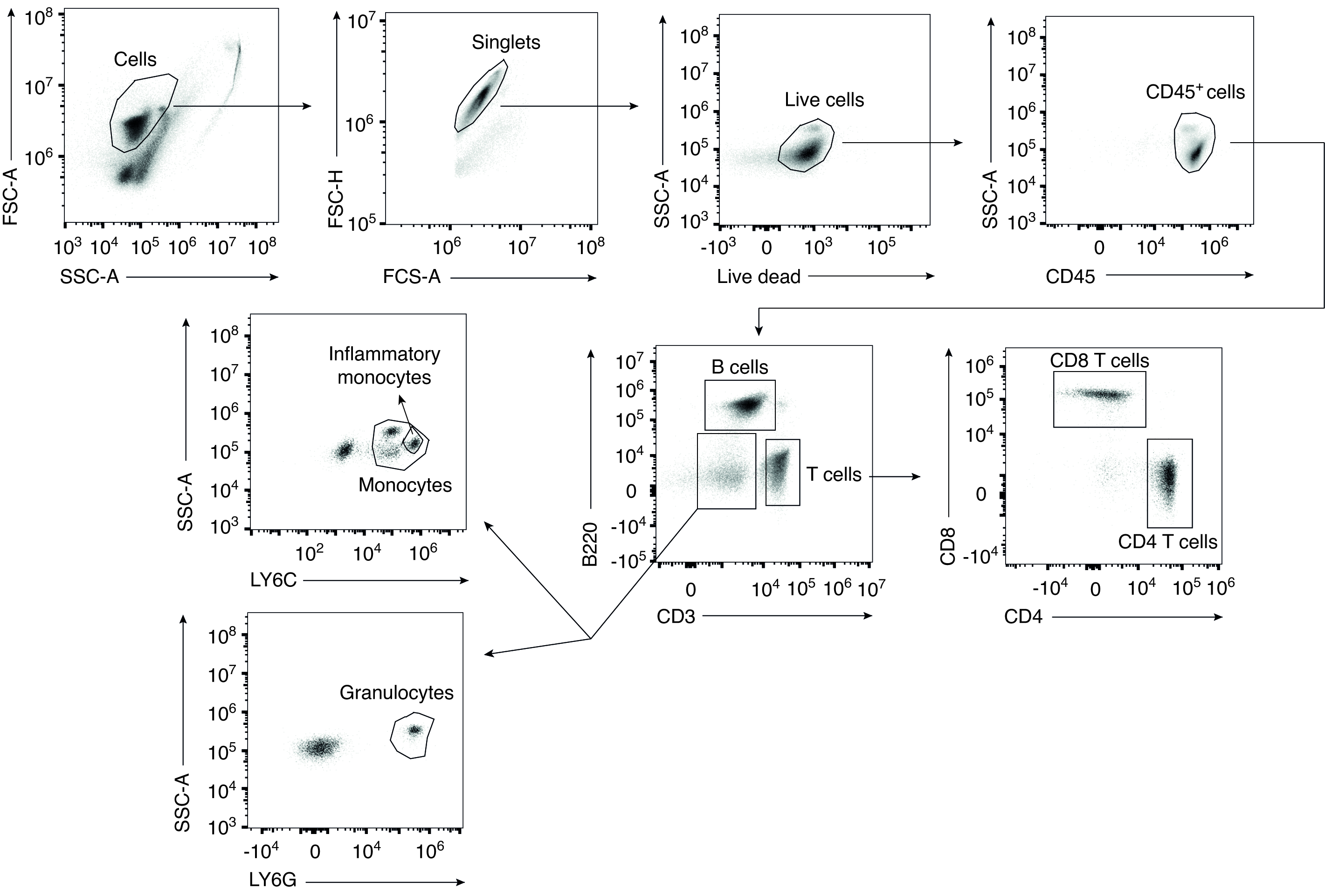

Supplement: Supplementary file 3 — Additional file 3: Supplemental Figure 3. Flow cytometry gating strategy for circulating immune cell populations study. B cells (CD45+ CD3- B220+), T cells (CD45+ CD3+ B220-), CD4+, CD8+ (CD45+ B220- CD3+), monocytes (CD45+ CD3- B220- Ly6C+), inflammatory monocytes (CD45+ CD3- B220- Ly6C+ high), and granulocytes (CD45+ CD3- B220- Ly6G+) were identified. [file 12979_2023_402_MOESM3_ESM.tif]

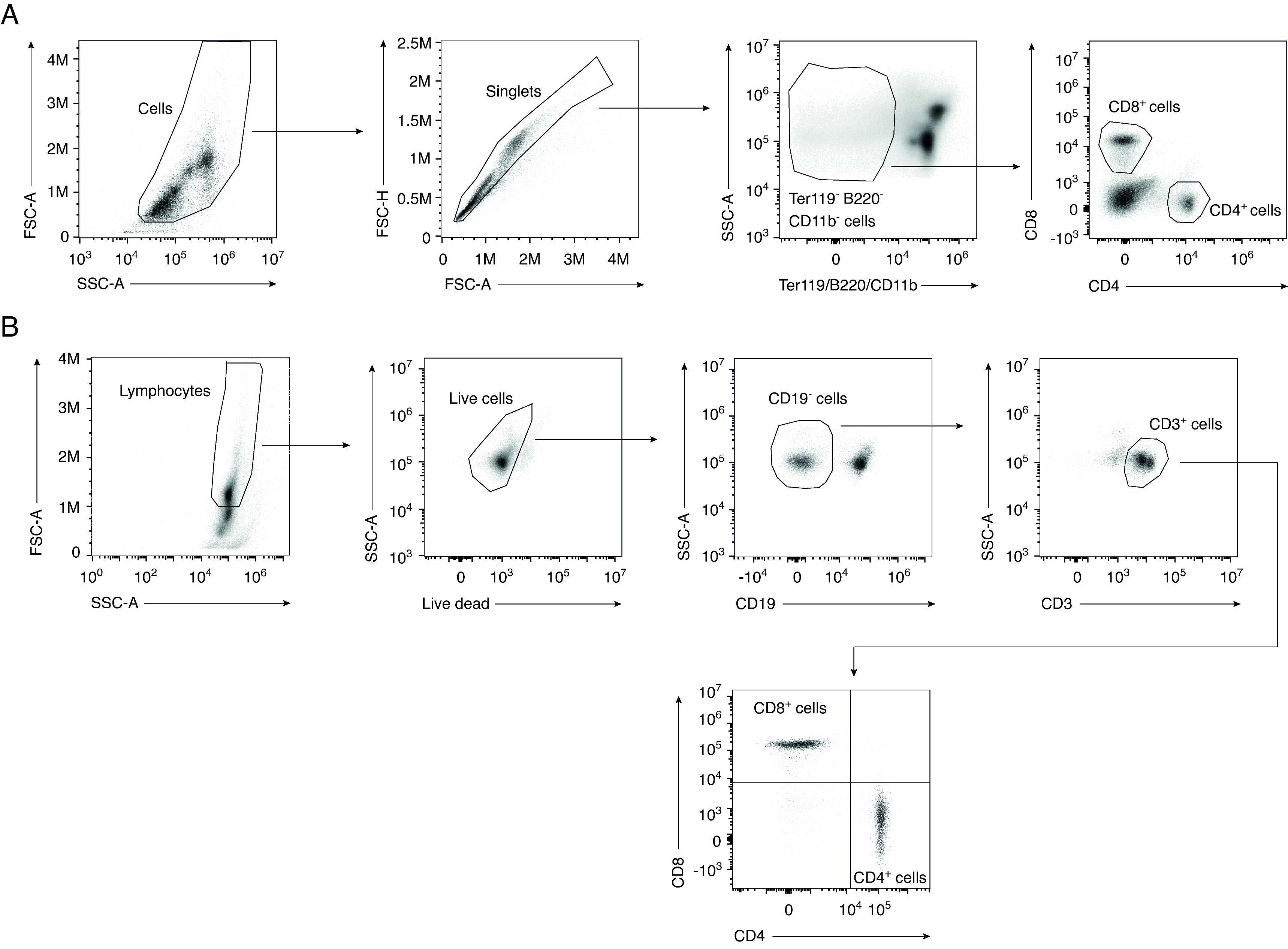

Supplement: Supplementary file 4 — Additional file 4: Supplemental Figure 4. Flow cytometry gating strategy for analysis of CD4+ and CD8+ cells in bone marrow and spleen. Gating strategies used to identify CD4+ and CD8+ cells in bone marrow (A) and spleen (B). [file 12979_2023_402_MOESM4_ESM.tif]

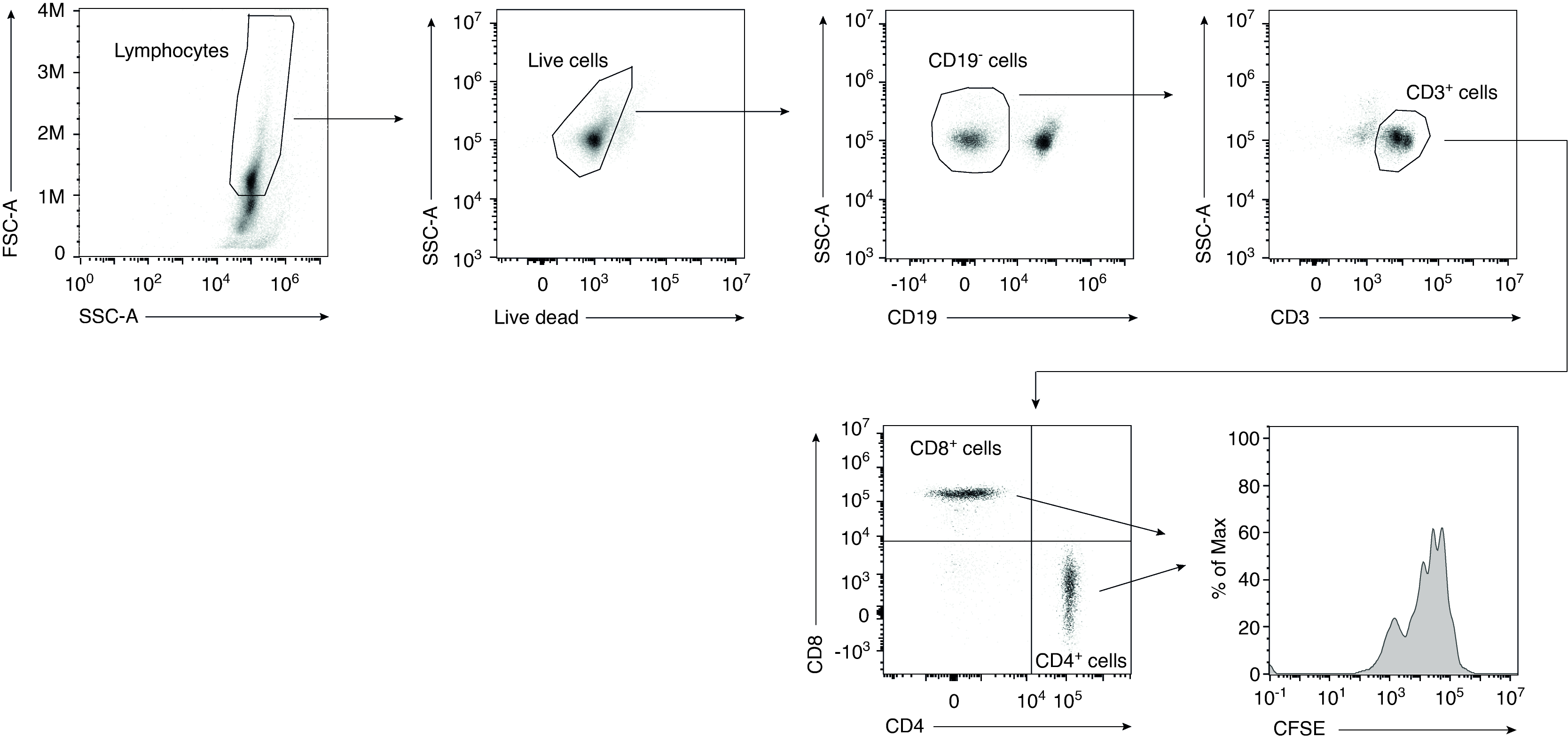

Supplement: Supplementary file 6 — Additional file 6: Supplemental Figure 6. Flow cytometry gating strategy for analysis of CD4+ and CD8+ proliferation. CD4+ and CD8+ splenocytes were identified and the dilution of the CFSE marker was assessed. [file 12979_2023_402_MOESM6_ESM.tif]

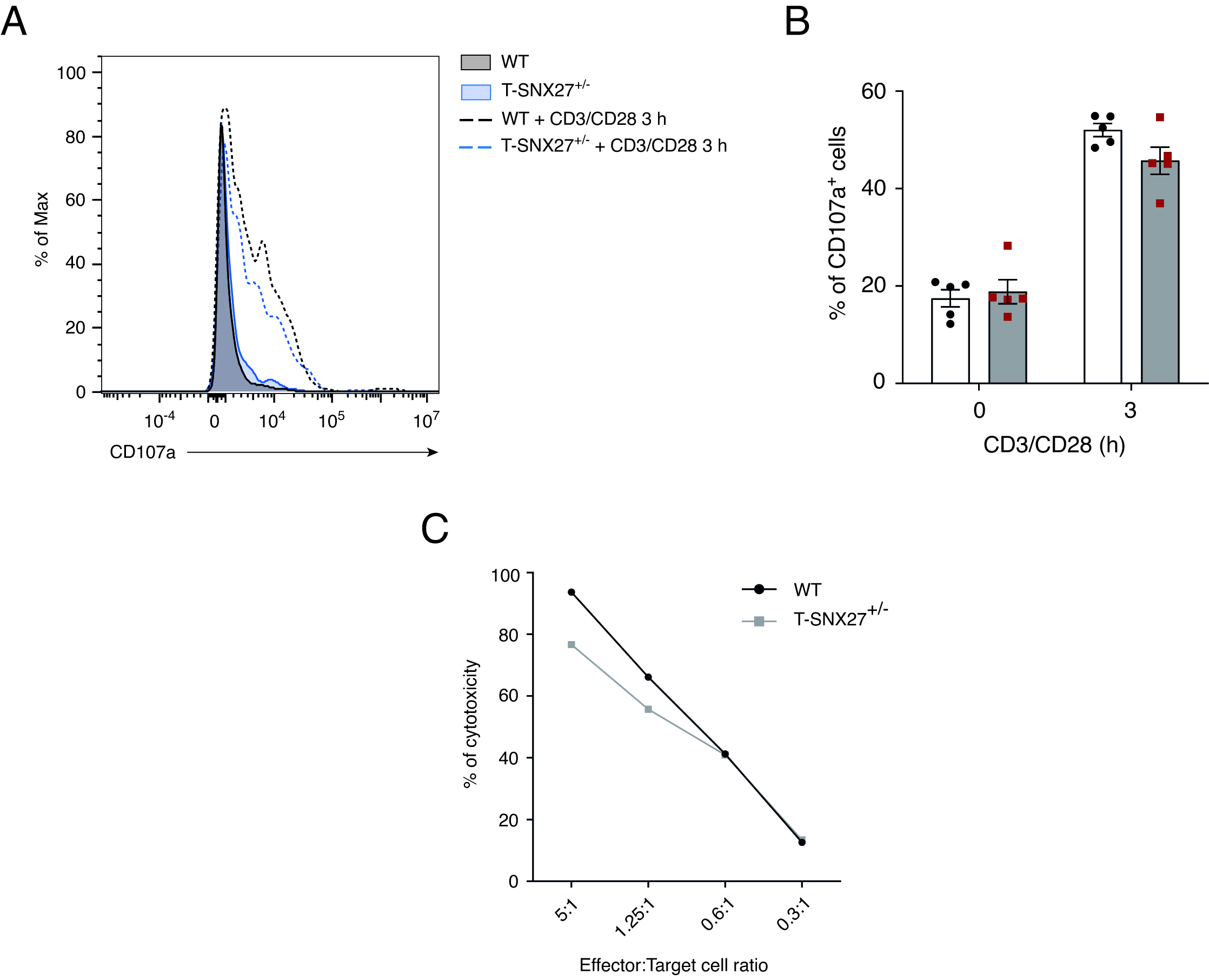

Supplement: Supplementary file 7 — Additional file 7: Supplemental Figure 7. Absence of SNX27 does not affect CTL LAMP-1 expression nor killing capacity. (A) Ex vivo-differentiated CTL from SNX27fl/flor CD4‑Cre‑SNX27fl/+ mice were stimulated for 3 h with plate‑bound anti-CD3 (5 μg/ml) and soluble anti-CD28 (2.5 μg/ml) in the presence of CD107a-PE (LAMP1) (2.5 μg/ml). Afterwards, surface analysis of CD107a was analyzed by flow cytometry. A representative flow cytometry plot is shown. (B) GMFI of surface CD107a. Data are shown as mean ± SEM; ns p>0.05; two-way ANOVA with Bonferroni post-test was used for multiple comparisons; n=5 mice. (C) Ex vivo‑differentiated CTL from SNX27fl/flor CD4‑Cre‑SNX27fl/+ mice were mixed with anti-CD3-coated P815 target cells (2.5 μg/ml) for 4 h at the depicted CTL/target cell ratios. Killing was examined by a colorimetric assay, as described in the methods section. A representative experiment is shown (n >3 mice). SNX: Sorting nexin; CTL: Cytotoxic T lymphocyte; GMFI: geometric mean fluorescence intensity; SEM: Standard error of the mean. [file 12979_2023_402_MOESM7_ESM.tif]
